# CD180-Associated B-Cell Alterations in Type 1 Diabetes and the In Vitro Effects of Chlorogenic Acid: An Integrated Transcriptomic and Experimental Study

**DOI:** 10.3390/ijms27198933

**Published:** 2026-10-08

**Authors:** Zihang Wang, Arzu Mijit, Rong Han, Mengjia Li, Yang Wu, Lufeng Cheng

**Affiliations:** 1 School of Pharmacy, Xinjiang Medical University, Urumqi 830017, China; 2 Xinjiang Key Laboratory of Biopharmaceuticals and Medical Devices, Urumqi 830017, China; 3 Engineering Research Center of Xinjiang and Central Asian Medicine Resources, Ministry of Education, Urumqi 830017, China; 4 Xinjiang Key Laboratory of Natural Medicines Active Components and Drug Release Technology, Urumqi 830017, China

**Keywords:** type 1 diabetes, chlorogenic Acid, CD180, B cells, immune regulation

## Abstract

Type 1 diabetes mellitus (T1DM) is an autoimmune disease involving progressive loss of pancreatic β-cells. Although B cells have been implicated in T1DM progression, the molecular characteristics associated with disease-related B-cell states remain unclear. Here, we investigated B-cell-associated molecular alterations in T1DM and explored the potential immunomodulatory effects of a natural compound. Public T1DM transcriptomic datasets were analyzed using differential expression analysis, WGCNA, and machine-learning approaches. Single-cell RNA sequencing was used to characterize *Cd180* expression across B-cell states. Molecular docking and molecular dynamics simulations were used to explore potential interactions between CD180 and candidate compounds, and an exploratory DARTS assay was performed to assess CGA-associated changes in CD180 proteolytic susceptibility. CD180 expression was examined in pancreatic tissues from vehicle- and MSA-2-treated NOD mice. The effects of chlorogenic acid (CGA) were evaluated in vitro by exposing B cells to CGA during inflammatory stimulation, followed by washing and co-culture with MIN6 cells in CGA-free medium. Integrated transcriptomic analysis identified four T1DM-associated genes (*CD180*, *KCNJ2*, *STEAP4*, and *ZXDA*), among which *CD180* showed the strongest association with B-cell-related immune alterations. Single-cell analysis showed increased *Cd180* expression in inflammatory B-cell states together with enrichment of antigen-presentation-related programs. An exploratory DARTS experiment with matched loading-control data suggested altered CD180 proteolytic susceptibility following CGA exposure, although independent reproducibility remains to be established. In vitro, CGA exposure during inflammatory stimulation was associated with reduced CD180 expression in B cells and improved MIN6 cell viability and insulin-secretory responses during subsequent co-culture in CGA-free medium. CD180 is associated with inflammatory B-cell alterations in T1DM and may represent a candidate molecular feature of B-cell dysregulation. CGA treatment was associated with changes in CD180 expression in activated B cells and improved β-cell functional outcomes in vitro. These findings support further investigation of the relationship between CGA and CD180; however, whether the effects of CGA are specifically mediated through CD180 requires further experimental validation.

## 1. Introduction

Type 1 diabetes (T1DM) results from autoimmune destruction of pancreatic β-cells, which gradually reduces insulin production and leads to lifelong dependence on insulin therapy [1,2]. Although advances in glucose monitoring and insulin replacement have improved patient management, these treatments do not stop the autoimmune processes responsible for β-cell loss. Further investigation of immune regulation in T1DM is therefore needed to identify new strategies for preserving β-cell function.

Both T and B cells participate in the autoimmune response that damages pancreatic β-cells in T1DM [3,4]. T cells are major effectors of this injury, but B cells have functions that extend well beyond autoantibody production [5,6]. They can capture and present β-cell antigens to autoreactive T cells. B-cell-derived cytokines may also modify the surrounding immune environment. This functional contribution is supported by NOD mouse studies, in which B-cell depletion delays diabetes onset and impaired B-cell antigen presentation reduces autoreactive T-cell responses [7,8]. On this basis, we examined the molecular features associated with B-cell changes in T1DM.

CD180 is a leucine-rich repeat-containing receptor that is predominantly expressed in B cells and antigen-presenting cells [9]. Previous studies have shown that CD180 forms a complex with MD-1 and can modulate B-cell responses through TLR-associated signaling pathways [10,11]. However, whether CD180 contributes to autoimmune diabetes remains largely unknown.

Chlorogenic acid (CGA) is a plant-derived polyphenol that is present in many dietary sources [12]. It has been reported to possess anti-inflammatory and antioxidant properties, with additional effects on metabolic regulation [13]. CGA has also been reported to improve glucose metabolism and influence inflammatory responses [14]. However, its potential role in regulating autoimmune processes in T1DM, particularly B-cell-associated immune activation, has not been investigated.

We first analyzed T1DM transcriptomic datasets using machine-learning and network-based approaches to identify B-cell-related molecular changes. Single-cell RNA sequencing was then used to examine the distribution of *Cd180* across B-cell states. Based on these findings, we further investigated the potential association between CGA and CD180 using structural simulation and DARTS analysis. The effects of CGA were subsequently evaluated in vitro in a B cell–MIN6 β-cell co-culture system.

## 2. Results

### 2.1. Integrated Transcriptomic Analysis and WGCNA Identify T1DM-Associated Transcriptional Signatures

**Figure 1:**
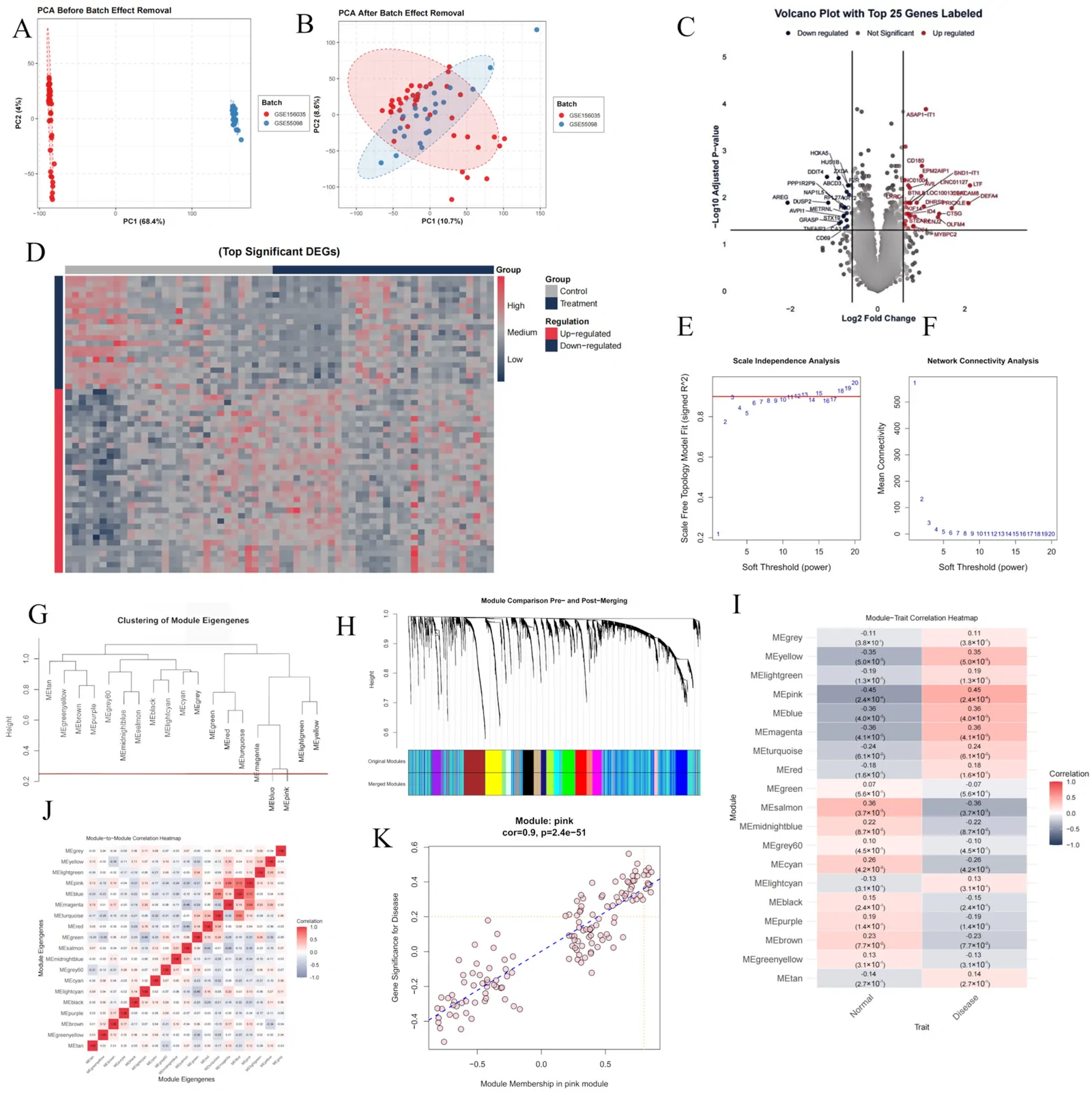
Transcriptomic integration, differential expression analysis, and weighted gene co-expression network analysis in T1DM. (**A**,**B**) PCA plots before (**A**) and after (**B**) batch correction. (**C**) Volcano plot showing 55 DEGs between T1DM patients and healthy controls, with the top 25 differentially expressed genes labeled. (**D**) Heatmap depicting the expression patterns of the top DEGs across the samples. (**E**,**F**) Analysis of the scale-free fit index (**E**) and mean connectivity (**F**) to determine the optimal soft-thresholding power (β = 8). (**G**,**H**) Clustering of module eigengenes (**G**) and the gene dendrogram with module assignment (**H**). (**I**) Heatmap showing the correlation between module eigengenes and clinical traits (Normal vs. Disease). (**J**) Heatmap illustrating the correlations among different modules. (**K**) Scatter plot showing the correlation between gene significance and module membership in the Pink module.

We first integrated the GSE156035 and GSE55098 datasets to examine transcriptional differences associated with T1DM. PCA showed a marked dataset-dependent separation before batch correction. This separation was substantially reduced after correction, with samples from the two datasets showing greater overlap (Figure 1A,B).

Differential expression analysis identified 55 genes that differed significantly between T1DM and control samples (Figure 1C,D). We next used WGCNA to examine co-expression patterns in the corrected expression matrix. A soft-thresholding power of β = 8 was selected according to the scale-free topology criterion, resulting in 33 co-expression modules (Figure 1E–H).

Among these modules, the Pink module showed the strongest association with T1DM status (Figure 1I). Within this module, gene significance was strongly correlated with module membership (cor = 0.90, *p* = 2.4 × 10^−51^; Figure 1J,K), indicating that genes more closely connected to the module were also more strongly associated with T1DM status.

### 2.2. Identification and Functional Characterization of Initial Candidate Genes

**Figure 2:**
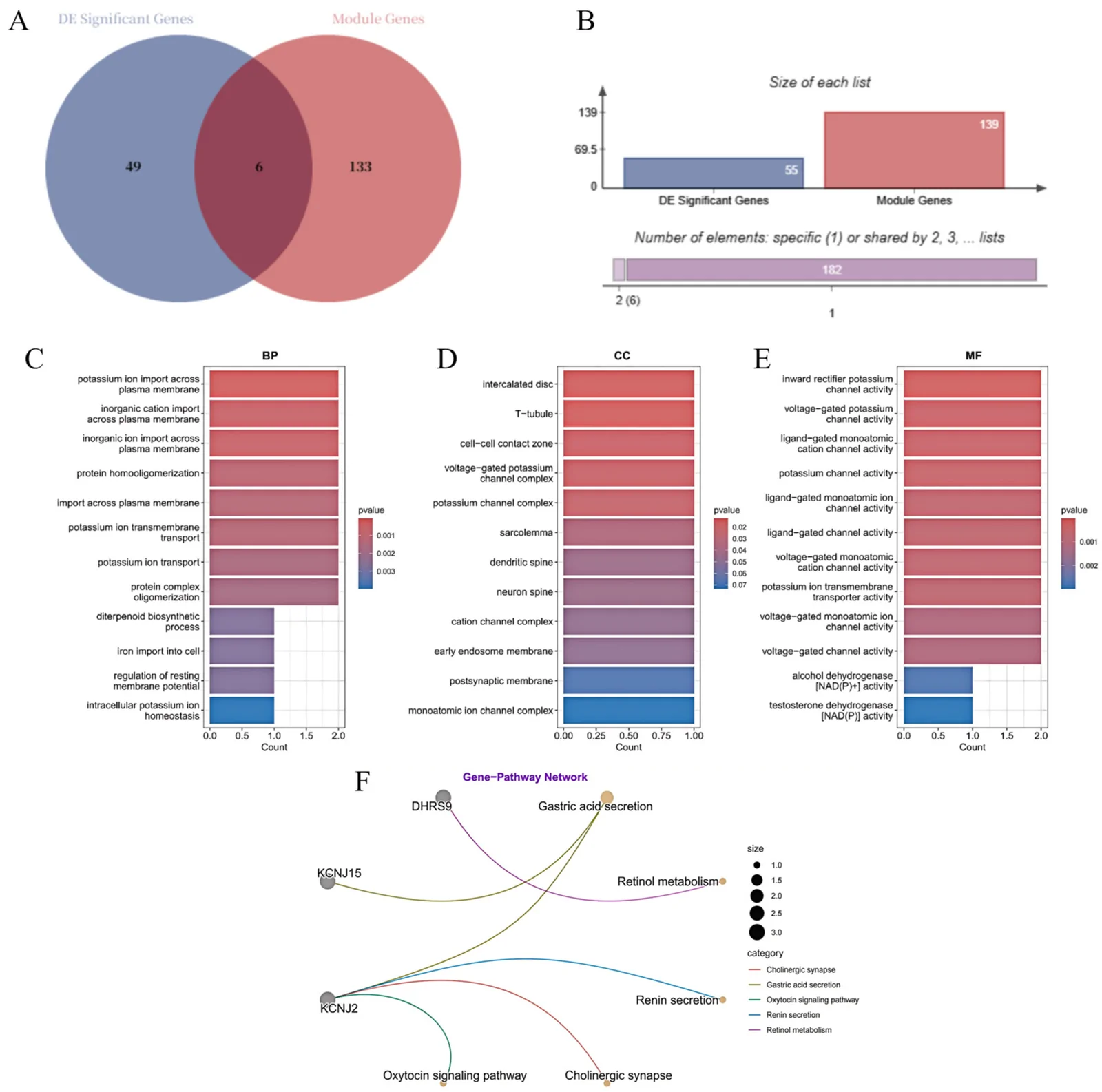
Screening and functional enrichment analysis of the initial candidate genes identified by WGCNA and differential expression analysis. (**A**) Venn diagram showing the intersection between genes in the T1DM-associated Pink module and T1DM-related differentially expressed genes, yielding six shared candidate genes. (**B**) UpSet plot further illustrating the intersection pattern of the candidate-gene sets. (**C**–**E**) GO enrichment analyses of the six overlapping genes in the biological process (BP), cellular component (CC), and molecular function (MF) categories, respectively. (**F**) KEGG pathway enrichment analysis of the six overlapping genes.

Intersection analysis between genes in the T1DM-associated Pink module and the differentially expressed genes (DEGs) identified six shared genes, which were defined as the initial candidate genes for subsequent analysis (Figure 2A,B). Functional enrichment analysis of these six genes revealed associations with arachidonic acid metabolism, extracellular matrix organization, extracellular matrix components, and collagen-related functions in GO analysis (Figure 2C–E). KEGG analysis further showed enrichment in arachidonic acid metabolism, the renin–angiotensin system, and cytokine–cytokine receptor interaction pathways (Figure 2F). These findings indicate that the six initial candidate genes are associated with inflammation- and extracellular matrix-related biological processes. The six genes were subsequently subjected to machine-learning-based feature selection to further prioritize T1DM-associated candidate genes.

### 2.3. Machine-Learning-Based Prioritization and Evaluation of T1DM-Associated Candidate Genes

**Figure 3:**
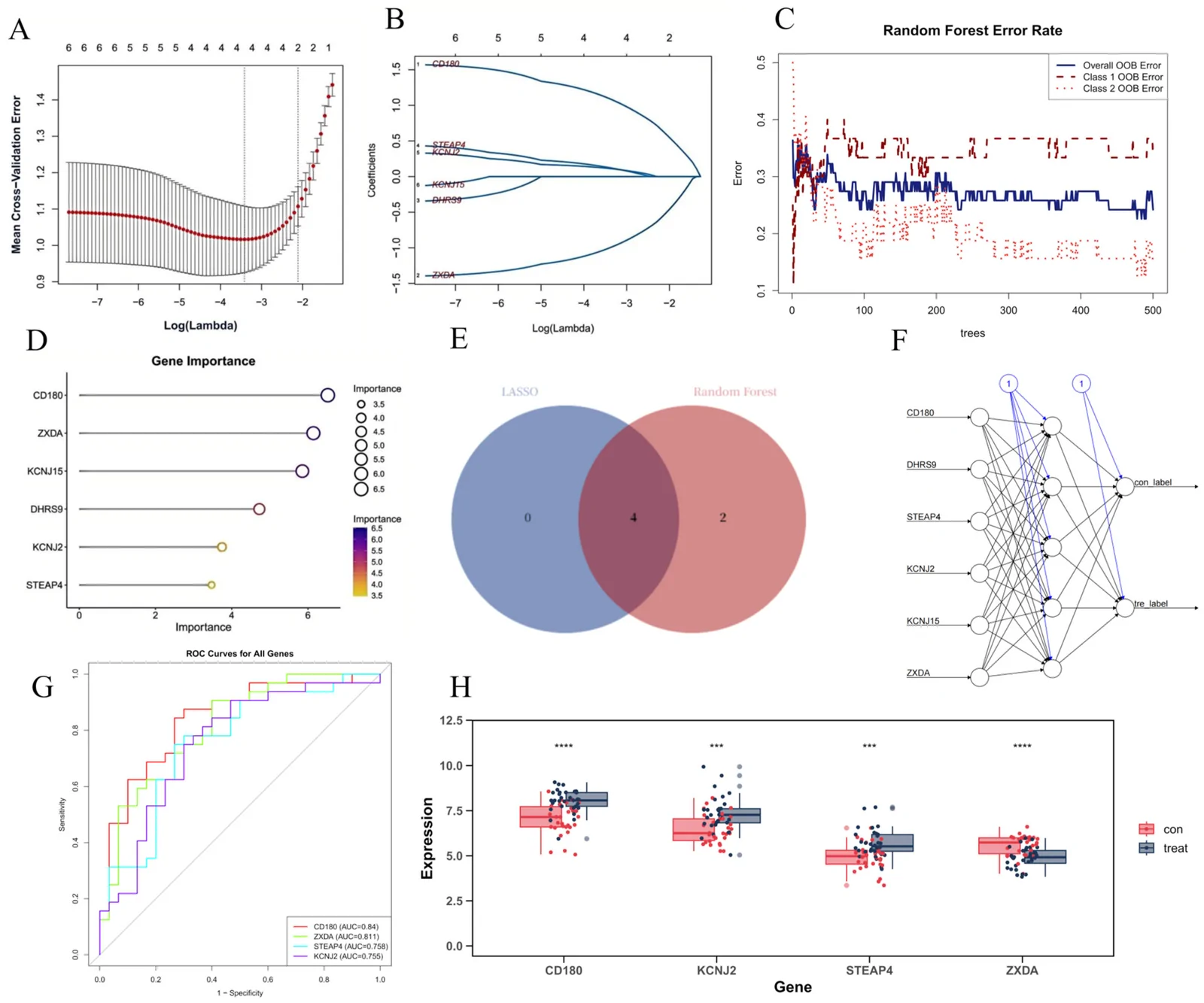
Machine-learning-based prioritization and evaluation of T1DM-associated candidate genes. (**A**) LASSO regression cross-validation curve for selection of the regularization parameter (λ = 0.0329). (**B**) LASSO coefficient profile showing four genes with non-zero coefficients at the selected λ. (**C**) Random forest out-of-bag (OOB) error as a function of the number of trees. (**D**) Random forest variable-importance ranking of the six initial candidate genes identified by intersecting the T1DM-associated Pink module with the differentially expressed genes. (**E**) Venn diagram showing the intersection of genes prioritized by LASSO and random forest, identifying four shared candidate genes: *CD180*, *KCNJ2*, *STEAP4*, and *ZXDA*. (**F**) Architecture of the exploratory artificial neural network (ANN) classifier constructed using the four candidate genes. (**G**) ROC curves showing the apparent discriminatory performance of the four individual candidate genes within the discovery cohort, with AUC values of 0.840 for *CD180*, 0.811 for *ZXDA*, 0.758 for *STEAP4*, and 0.755 for *KCNJ2*. (**H**) Box plots showing the expression levels of *CD180*, *KCNJ2*, *STEAP4*, and *ZXDA* in T1DM and control samples. The ROC analyses shown in panel (**G**) were performed using the discovery cohort and therefore represent apparent discrimination rather than independent validation. Internal assessment of the prespecified four-gene model is presented in Supplementary Figure S1. *** *p* < 0.001, **** *p* < 0.0001.

The six initial candidate genes identified by intersecting the T1DM-associated Pink module with the differentially expressed genes were subsequently subjected to machine-learning-based feature selection. LASSO regression retained four genes with non-zero coefficients at λ = 0.0329 (Figure 3A,B). Random forest analysis was subsequently used to assess the relative importance of the six initial candidate genes. The out-of-bag (OOB) error decreased as the number of trees increased and subsequently reached a stable level (Figure 3C), with CD180 showing the highest variable importance score (Figure 3D). Integration of the LASSO and random forest results identified four shared candidate genes: *CD180*, *KCNJ2*, *STEAP4*, and *ZXDA* (Figure 3E).

An exploratory artificial neural network (ANN) model was subsequently constructed using these four candidate genes (Figure 3F). Within the discovery cohort, single-gene ROC analysis showed apparent AUC values of 0.840 for *CD180*, 0.811 for *ZXDA*, 0.758 for *STEAP4*, and 0.755 for *KCNJ2* (Figure 3G). Expression analysis further showed significant differences in the expression of all four genes between T1DM and control samples (Figure 3H).

The stability of candidate-gene selection and the discriminatory performance of the four-gene signature were further examined using resampling-based internal assessment (Figure S1). Bootstrap LASSO analysis showed that *CD180* and *ZXDA* were the most consistently selected candidate genes, whereas *STEAP4* and *KCNJ2* showed comparatively lower selection frequencies (Figure S1D). For the prespecified four-gene logistic regression model, the apparent AUC was 0.883, whereas the summary ROC curve based on repeated stratified 10-fold cross-validated held-out predictions yielded an AUC of 0.827 (Figure S1A). Bootstrap analysis estimated a mean AUC optimism of 0.032 (Figure S1B). Cross-validated predictions yielded a Brier score of 0.174, and the calibration analysis showed overall agreement between predicted and observed probabilities, although some deviation was observed at intermediate predicted probabilities (Figure S1C). These findings provide conditional internal support for the stability and discriminatory performance of the four-gene model but do not constitute independent external validation.

### 2.4. Association Between Hub Genes and Immune Cell Infiltration Patterns

Immune cell proportions were estimated using CIBERSORT based on transcriptomic profiles. Correlation analysis was then performed to evaluate relationships between candidate genes and immune populations.

**Figure 4:**
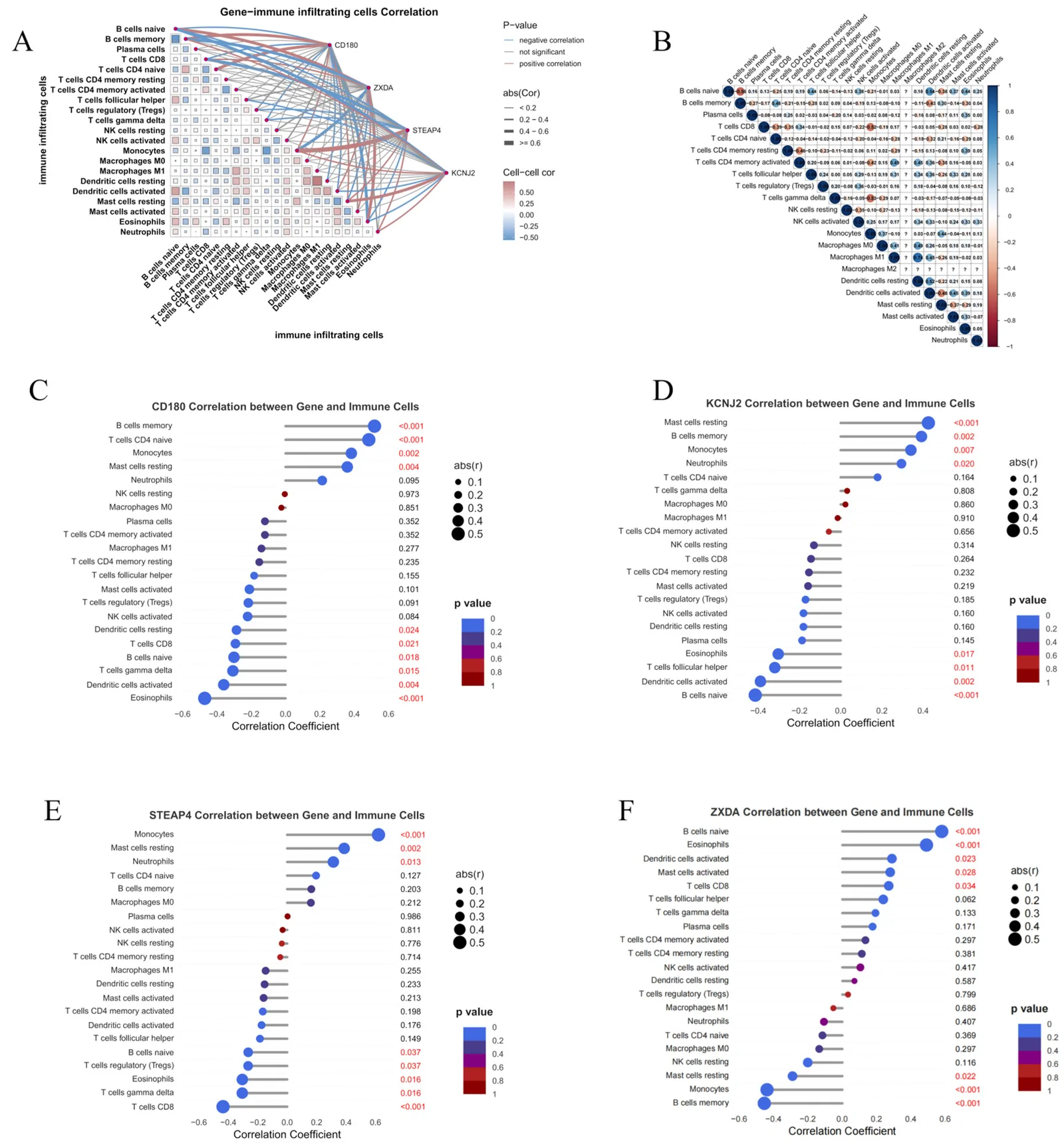
Correlation between hub genes and immune cell infiltration in T1DM. (**A**) Gene-immune cell correlation network showing the associations between four hub genes and distinct immune cell subsets. (**B**) Correlation heatmap illustrating the pairwise relationships between immune cell types. (**C**–**F**) Lollipop plots depicting the detailed correlation coefficients between the expression levels of *CD180* (**C**), *KCNJ2* (**D**), *STEAP4* (**E**), and *ZXDA* (**F**) and multiple immune infiltrating cells. The color scale indicates the *p*-value, and dot size represents the absolute correlation coefficient (|r|).

The four genes showed distinct immune association patterns (Figure 4A). *CD180* was positively correlated with memory B cells and negatively correlated with eosinophils (Figure 4C). *KCNJ2* was associated with resting mast cells and naive B cells, while *STEAP4* showed correlations with monocytes and M0 macrophages. *ZXDA* was related to both naive and memory B-cell populations (Figure 4D–F).

Notably, *CD180* showed a specific association with memory B-cell estimates among the candidate genes.

### 2.5. Evaluation of Diagnostic Performance and Functional Association of CD180 in T1DM

**Figure 5:**
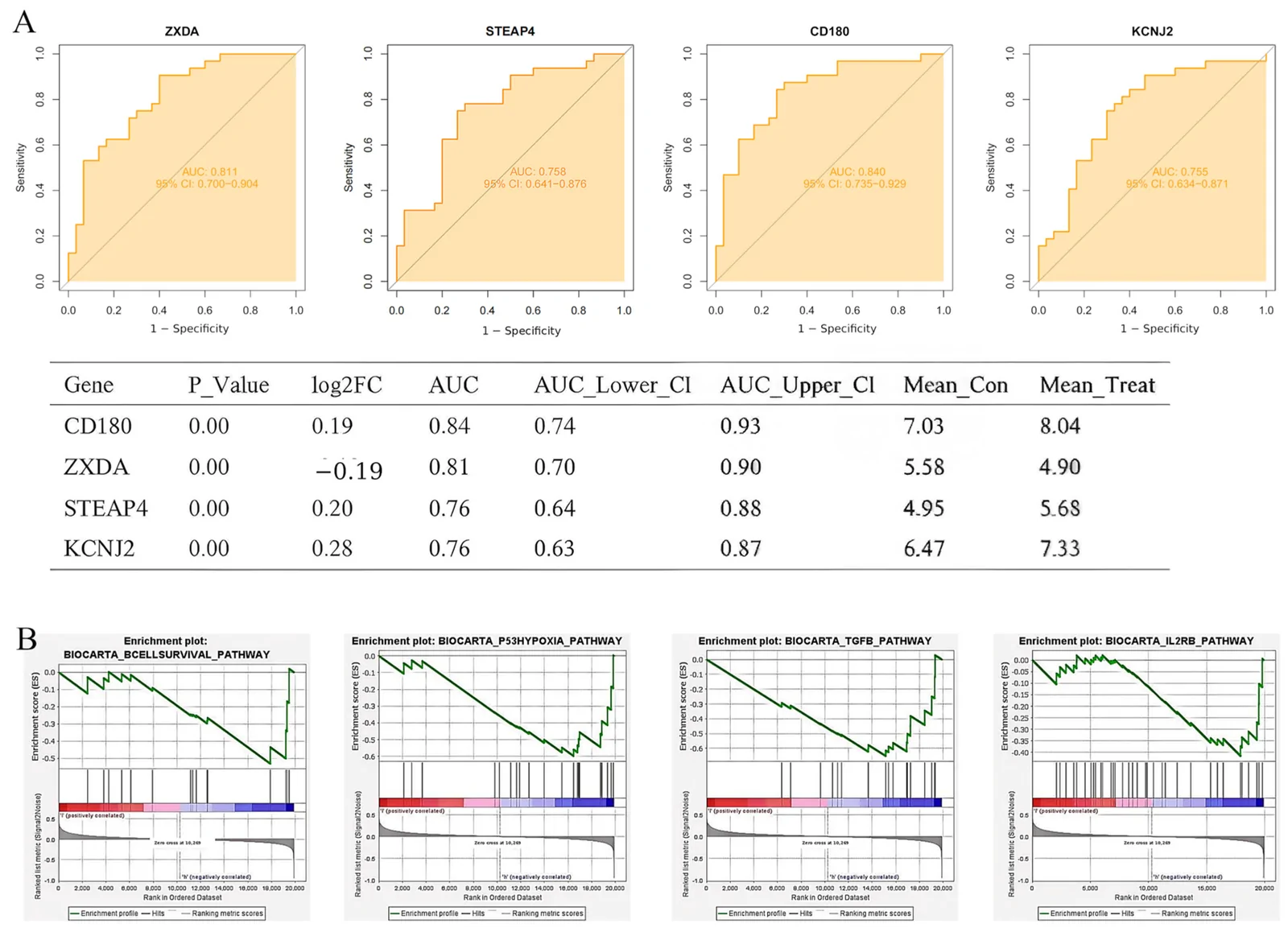
Classification performance of hub genes and *CD180*-associated pathway enrichment analysis. (**A**) ROC curves of the four candidate genes with their AUC values. (**B**) GSEA plots showing pathways enriched among *CD180*-associated genes.

We compared the ability of the four candidate genes to classify T1DM and control samples using ROC analysis. *CD180* achieved the highest AUC among the tested genes (0.840), whereas *ZXDA*, *STEAP4*, and *KCNJ2* showed AUC values of 0.811, 0.758, and 0.755, respectively (Figure 5A).

Because *CD180* showed a strong association with T1DM classification, we analyzed genes correlated with *CD180* expression by GSEA. The enriched pathways included B-cell survival, hypoxia response, TGF-β signaling, and IL-1 receptor-related signaling (Figure 5B). These findings suggest that *CD180* expression is associated with transcriptional programs involved in B-cell-related inflammatory responses.

### 2.6. Transcriptomic Profiling Identifies Cd180-Associated Changes in Activated B Cells

We further tested whether *Cd180* expression changed during inflammatory activation of B cells. RNA-seq was performed on B cells collected from control and stimulated conditions in the co-culture system.

**Figure 6:**
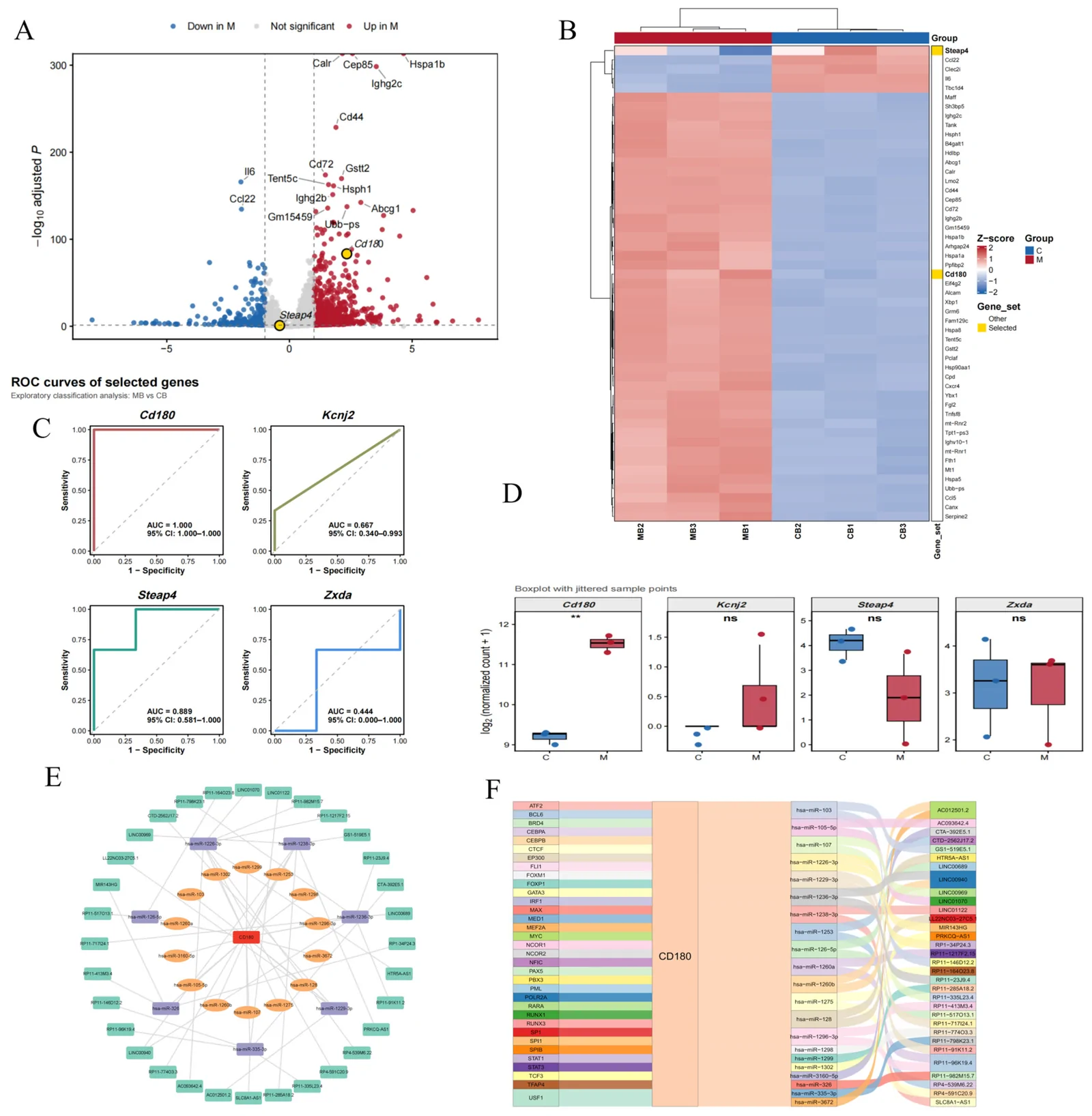
Transcriptomic profiling of B cells following co-culture with MIN6 cells and prediction of CD180-associated regulatory networks. (**A**) Volcano plot showing differentially expressed genes between the model (M) and control (C) groups, identified using DESeq2. Genes with |log_2_ fold change| ≥ 1 and Benjamini–Hochberg-adjusted *p* < 0.05 were considered differentially expressed. Red and blue points represent genes upregulated and downregulated in M relative to C, respectively; grey points indicate genes that did not meet these criteria. (**B**) Heatmap showing the expression patterns of selected differentially expressed genes across the six biological samples. Colors indicate standardized expression values (Z-scores). (**C**) Receiver operating characteristic (ROC) curves for *Cd180*, *Kcnj2*, *Steap4*, and *Zxda*. These exploratory analyses assess discrimination between M and C within the same RNA-seq dataset and do not constitute independent validation of diagnostic performance. (**D**) Box plots with individual sample points showing expression of the four candidate genes as log_2_(normalized count + 1). Each point represents one independent biological sample. Differences between the model (M) and control (C) groups were assessed separately for each gene using an unpaired two-tailed Student’s *t*-test. ** *p* < 0.01; ns, not significant, for M versus C. (**E**) Predicted competing endogenous RNA (ceRNA) network linking *CD180* with miRNAs and lncRNAs. (**F**) Sankey diagram summarizing predicted relationships among transcription factors, *CD180*, miRNAs, and lncRNAs. The networks in E and F represent computational predictions and have not been experimentally validated. Note: RNA-seq included three independent biological samples per group (*n* = 3; six samples in total). C: unstimulated B cells co-cultured with MIN6 cells; M: B cells stimulated with LPS, IFN-γ, and IL-1β before co-culture with MIN6 cells.

The two groups showed distinct transcriptional profiles. Differential expression analysis identified broad gene expression changes, and hierarchical clustering clearly separated control and stimulated samples (Figure 6A,B).

*Cd180* showed strong discrimination between the two groups, with an AUC of 1.000 in this dataset (Figure 6C). Consistent with the sequencing results, *Cd180* expression was increased after inflammatory stimulation, whereas the other three candidate genes showed no significant changes (Figure 6D).

Database-based TF and ceRNA prediction revealed several potential regulators associated with *Cd180*, including *Atf2*, *Bcl3*, *Cebpb*, *Ets1*, *Stat3*, *Tp53*, and *Miir17hg* (Figure 6E,F).

These data further highlight *Cd180* as the most consistently altered candidate gene in inflammatory B-cell conditions.

### 2.7. Single-Cell Transcriptomic Analysis Reveals Cd180-Associated B-Cell Inflammatory Characteristics

**Figure 7:**
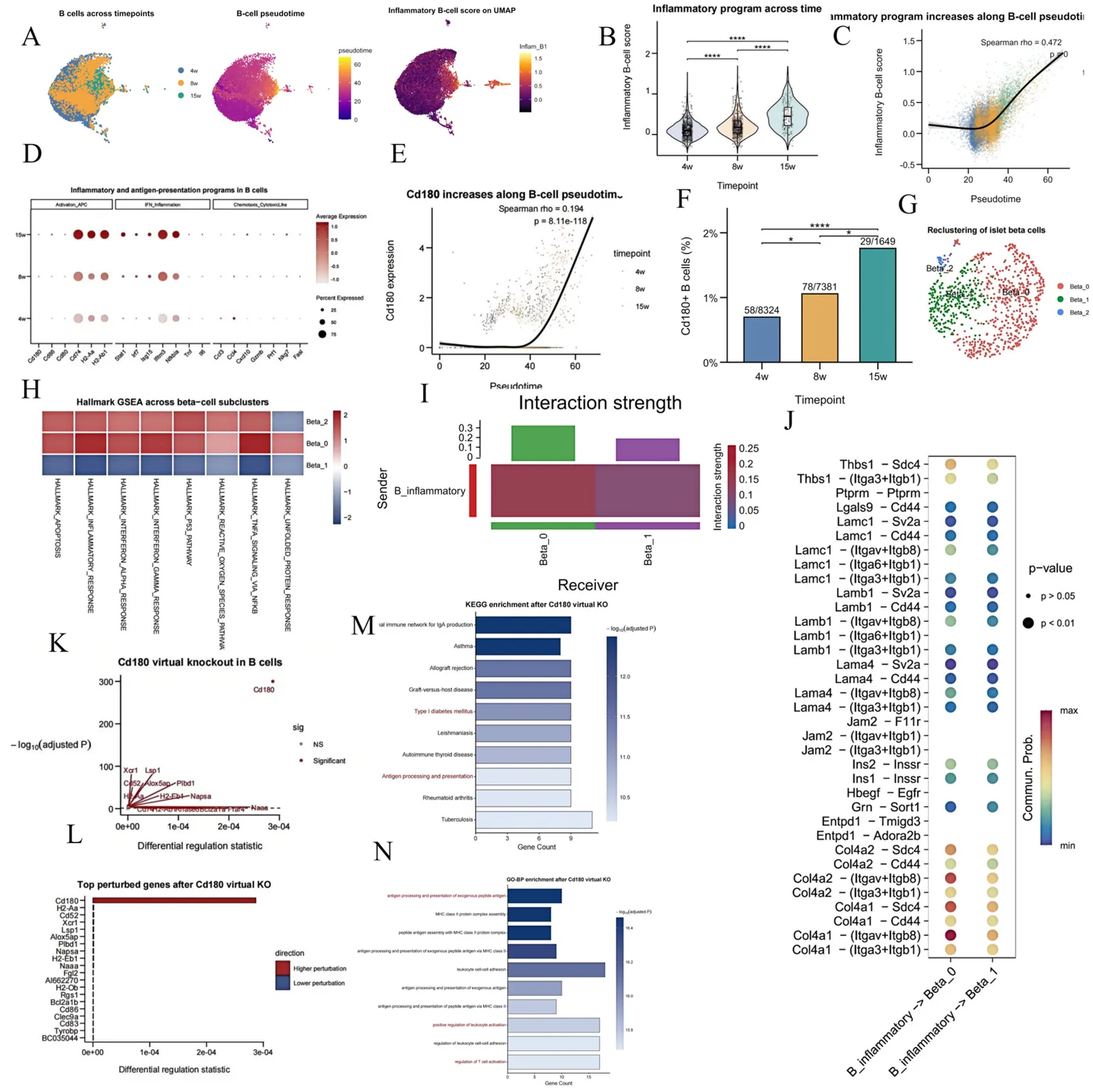
Single-cell analysis reveals *Cd180*-associated B-cell inflammatory features and immune–β-cell communication in T1DM. (**A**) UMAP projections of B cells across timepoints and the B-cell pseudotime trajectory. (**B**) UMAP visualization of the inflammatory B-cell score. (**C**) Violin plot showing the dynamic progression of the inflammatory program in B cells across different timepoints. (**D**) Scatter plot illustrating the positive correlation between the inflammatory B-cell score and pseudotime. (**E**) Dot plot depicting the expression profiles of genes involved in inflammatory responses and antigen presentation across distinct B-cell subclusters. (**F**) Scatter plot demonstrating the progressive increase in *Cd180* expression along B-cell pseudotime. (**G**) Relative proportion of *Cd180^+^* B-cell populations across different disease stages. (**H**) UMAP visualization of endocrine β-cell populations identified from the integrated single-cell dataset. (**I**) Heatmap illustrating the Hallmark Gene Set Enrichment Analysis (GSEA) results across the three beta-cell subclusters. (**J**) Heatmap displaying the interaction strength between inflammatory B cells and the islet beta-cell subclusters. (**K**) Dot plot showing the predicted probabilities of specific ligand–receptor interactions involved in cell–cell communication from inflammatory B cells to β-cell subclusters. (**L**) Scatter plot showing the differential regulation statistics of downstream genes following computational *Cd180* perturbation. (**M**) Bar plot ranking the top genes with the highest perturbations associated with computational *Cd180* perturbation. (**N**) Bar plots summarizing the KEGG pathway and GO functional enrichment analyses of the genes associated with computational *Cd180* perturbation. * *p* < 0.05, **** *p* < 0.0001.

We next used the GSE141786 dataset to examine B-cell states and their relationship with pancreatic β-cell populations. B-cell clustering and pseudotime analysis revealed a continuous shift in B-cell transcriptional states (Figure 7A).

B cells located along the inflammatory trajectory showed higher inflammatory scores and different expression patterns of antigen presentation-related genes (Figure 7B–D). *Cd180* expression followed a similar pattern, increasing with the transition toward inflammation-associated B-cell states (Figure 7E,F).

Three β-cell populations were identified from the same dataset. These subclusters displayed different pathway activities related to metabolism, stress responses, apoptosis, and glycolysis (Figure 7G,H). Predicted ligand–receptor analysis revealed several potential interactions between inflammatory B cells and β-cell populations (Figure 7I,J).

We further applied scTenifoldKnk to assess transcriptomic changes associated with in silico *Cd180* perturbation. Genes identified in association with computational *Cd180* knockout included *Sftpd*, *B2m*, *Cd74*, and *H2-Ab1*, with enrichment in antigen-presentation and T-cell-related immune pathways (Figure 7K–N).

These data indicate that *Cd180* is associated with a specific inflammatory B-cell transcriptional state characterized by antigen presentation-related features.

### 2.8. Phenotypic and Immune-Associated Changes in MSA-2-Treated NOD Mice

**Figure 8:**
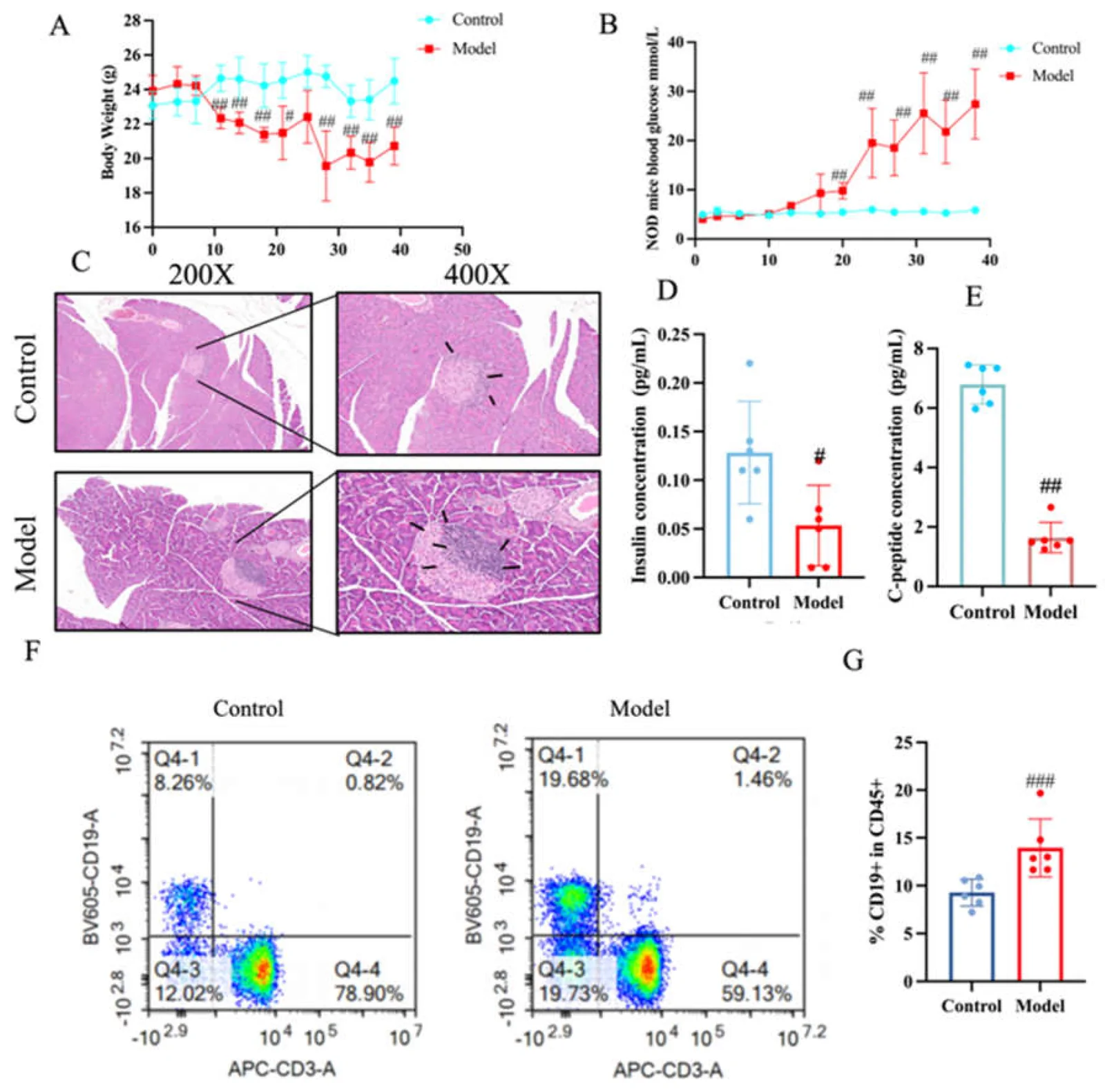
Phenotypic and immune-associated changes in MSA-2-treated NOD mice. (**A**) Changes in body weight in vehicle- and MSA-2-treated NOD mice over the 40-day observation period. (**B**) Longitudinal changes in fasting blood glucose levels. (**C**) Representative H&E-stained pancreatic sections showing islet morphology and inflammatory cell infiltration (200× and 400× magnification; black arrows indicate inflammatory cell infiltration). (**D**,**E**) Serum levels of insulin (**D**) and C-peptide (**E**) measured by ELISA. (**F**,**G**) Flow cytometry gating strategy for identifying CD19^+^ B cells in peripheral blood lymphocytes (**F**), and statistical analysis of the percentage of CD19^+^ cells within the CD45^+^ population. Colors in the pseudocolor flow-cytometry plots indicate relative event density (**G**). Control: vehicle-treated NOD mice; Model: MSA-2-treated NOD mice. Note: Data are presented as mean ± SD (*n* = 6 mice per group). Longitudinal changes in body weight and fasting blood glucose were analyzed using two-way repeated-measures ANOVA followed by Šídák’s multiple-comparisons test. Serum insulin and C-peptide concentrations and the proportion of CD19^+^ cells within the CD45^+^ population were analyzed using an unpaired two-tailed Student’s *t*-test. (^#^ *p* < 0.05, ^##^ *p* < 0.01, ^###^ *p* < 0.001, compared with the Control group).

We compared metabolic and immune-associated parameters between vehicle- and MSA-2-treated NOD mice over a 40-day observation period. Compared with vehicle-treated mice, MSA-2-treated mice showed lower body weight and higher fasting blood glucose levels (Figure 8A,B). Pancreatic sections from MSA-2-treated mice exhibited increased inflammatory infiltration and disruption of islet architecture (Figure 8C). Serum insulin and C-peptide levels were also reduced in MSA-2-treated mice compared with the vehicle-treated group (Figure 8D,E). Analysis of peripheral blood lymphocytes further showed an increased proportion of CD19^+^ B cells in MSA-2-treated mice (Figure 8F,G). Additional flow-cytometry plots and corresponding event-count tables are provided in Supplementary (Figure S2).

### 2.9. Increased CD180 Expression in T1DM Models and Effects of Activated B Cells on β-Cell Function

**Figure 9:**
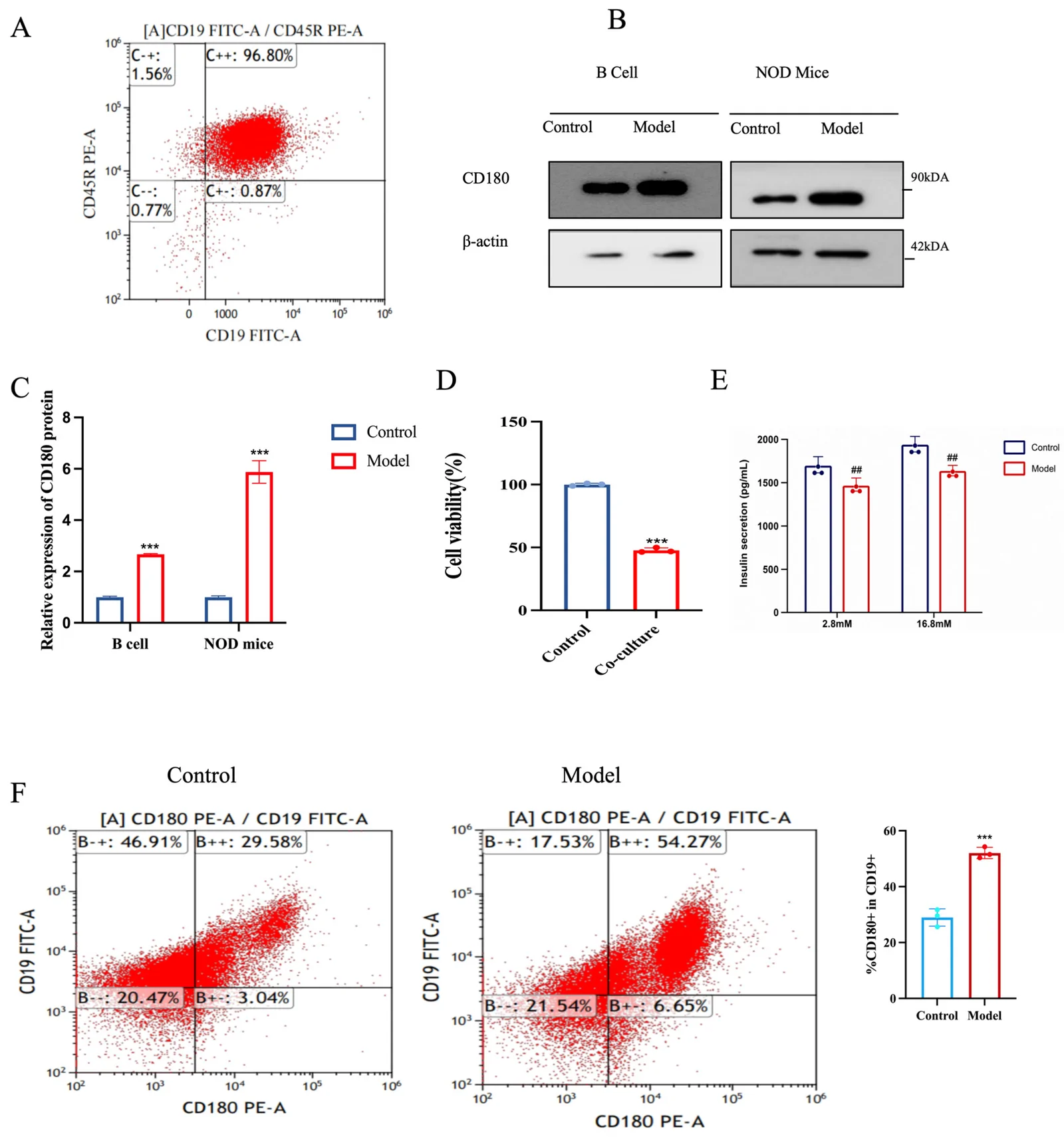
Assessment of B-cell purity, CD180 expression, and effects of B-cell co-culture on β-cell function. (**A**) Flow cytometric analysis confirming the purity of isolated primary B cells by gating on CD19^+^ and CD45R^+^ populations. (**B**) Western blot analysis of CD180 protein expression in isolated B cells and pancreatic tissues from vehicle- and MSA-2-treated NOD mice. (**C**) Quantification of CD180 protein expression. (**D**) Cell viability of MIN6 cells analyzed by CCK-8 assay in the Control and Co-culture groups. (**E**) GSIS analysis under low- and high-glucose stimulation conditions. (**F**) Representative flow-cytometry plots and statistical bar chart analyzing the percentage of CD180^+^ cells within the CD19^+^ B cell population in the co-culture system. Note: Data are presented as mean ± SD. (*** *p* < 0.001 vs. Control; ## *p* < 0.01 vs. Control), *n* = 3. Quantitative analyses of Western blotting, CCK-8, and flow cytometry were performed using three independent biological replicates (*n* = 3). Comparisons between two groups were performed using an unpaired two-tailed Student’s *t*-test. Statistical significance is indicated as shown in the figure. At each glucose concentration, comparisons between the Control and Model groups were performed using an unpaired two-tailed Student’s *t*-test.

We first examined CD180 expression in B cells exposed to inflammatory stimulation. The isolated cell preparation showed a CD19^+^ CD45R^+^ fraction of 96.8% within the FSC/SSC-selected population in the illustrated sample (Figure 9A and Figure S3A).

CD180 protein abundance was higher in inflammation-stimulated B cells than in unstimulated B cells. In the animal experiment, whole-pancreas lysates from MSA-2-treated NOD mice showed higher CD180 abundance than those from vehicle-treated NOD mice (Figure 9B,C).

To determine whether inflammatory B-cell changes were associated with β-cell impairment, stimulated B cells were co-cultured with MIN6 cells. MIN6 viability decreased after co-culture, and insulin secretion responses to both low and high glucose stimulation were reduced (Figure 9D,E).

Flow cytometry showed an increased frequency of CD180^+^ cells among CD19^+^ B cells following stimulation (Figure 9F; Supplementary Figure S3B). These experiments showed increased CD180 expression under inflammatory conditions and altered MIN6 responses in the co-culture system.

### 2.10. Identification of Chlorogenic Acid as a Candidate Compound Through Structure-Based Screening

**Figure 10:**
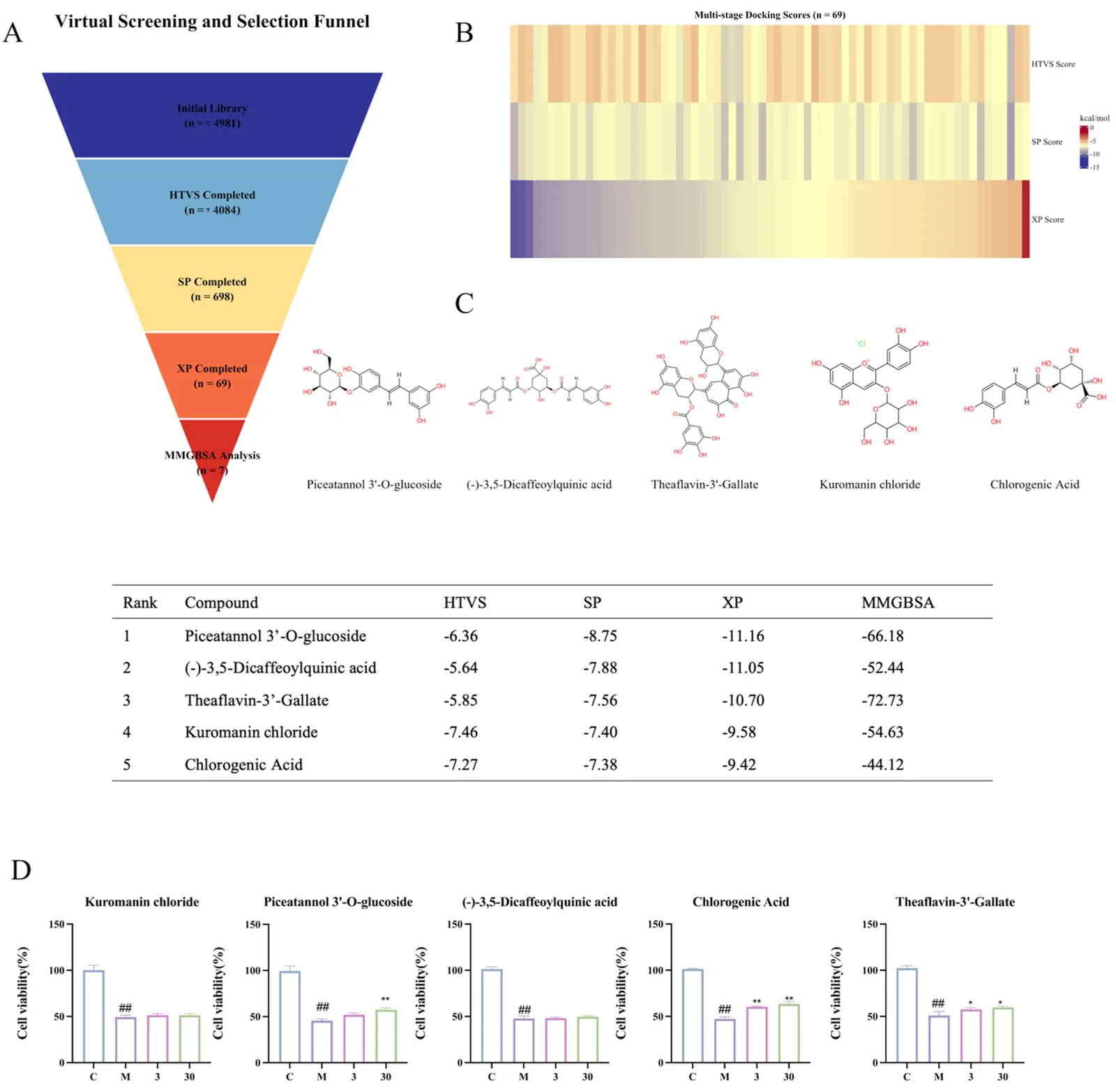
Structure-based virtual screening and functional evaluation of candidate compounds. (**A**) Virtual screening funnel showing the sequential selection process from the initial compound library (*n* = 4981) through HTVS, SP, and XP docking. Among the 69 compounds selected after XP docking, 7 compounds were subjected to MM/GBSA analysis, and 5 compounds were subsequently selected for experimental evaluation based on predicted affinity and compound availability. (**B**) HTVS, SP, and XP for the 69 compounds selected after the XP docking stage. (**C**) Chemical structures of the top 5 candidate compounds (Piceatannol 3’-O-glucoside, (-)-3,5-Dicaffeoylquinic acid, Theaflavin-3′-Gallate, Kuromanin chloride, and Chlorogenic Acid), and the summary table detailing their ranking, docking scores, and MMGBSA binding free energies. (**D**) CCK-8 assays evaluating the effects of the five candidate compounds on MIN6 cell viability following exposure to activated B cells. Cells were treated with 3 μM or 30 μM of each compound. C, Control group; M, Model group. Data are presented as mean ± SD from three independent biological replicates (*n* = 3). Statistical differences among groups were analyzed using one-way ANOVA followed by Dunnett’s multiple-comparisons test. (## *p* < 0.01 vs. Control group; * *p* < 0.05, ** *p* < 0.01 vs. Model group).

We screened small molecules against CD180 using a structure-based docking approach. From the initial library of 4981 compounds, HTVS, SP, and XP docking identified 69 candidates for further MM/GBSA analysis (Figure 10A,B).

Five compounds were chosen based on their docking and energy profiles and were tested experimentally. These compounds included Piceatannol 3’-O-glucoside, (-)-3,5-Dicaffeoylquinic acid, Theaflavin-3′-Gallate, Kuromanin chloride, and CGA (Figure 10C).

When tested in the B cell–MIN6 co-culture system, CGA showed the strongest protective effect on MIN6 viability among the five compounds at 30 μM (Figure 10D). We therefore selected CGA for further investigation.

### 2.11. Structural Analysis and DARTS Evaluation of the CGA–CD180 Association

**Figure 11:**
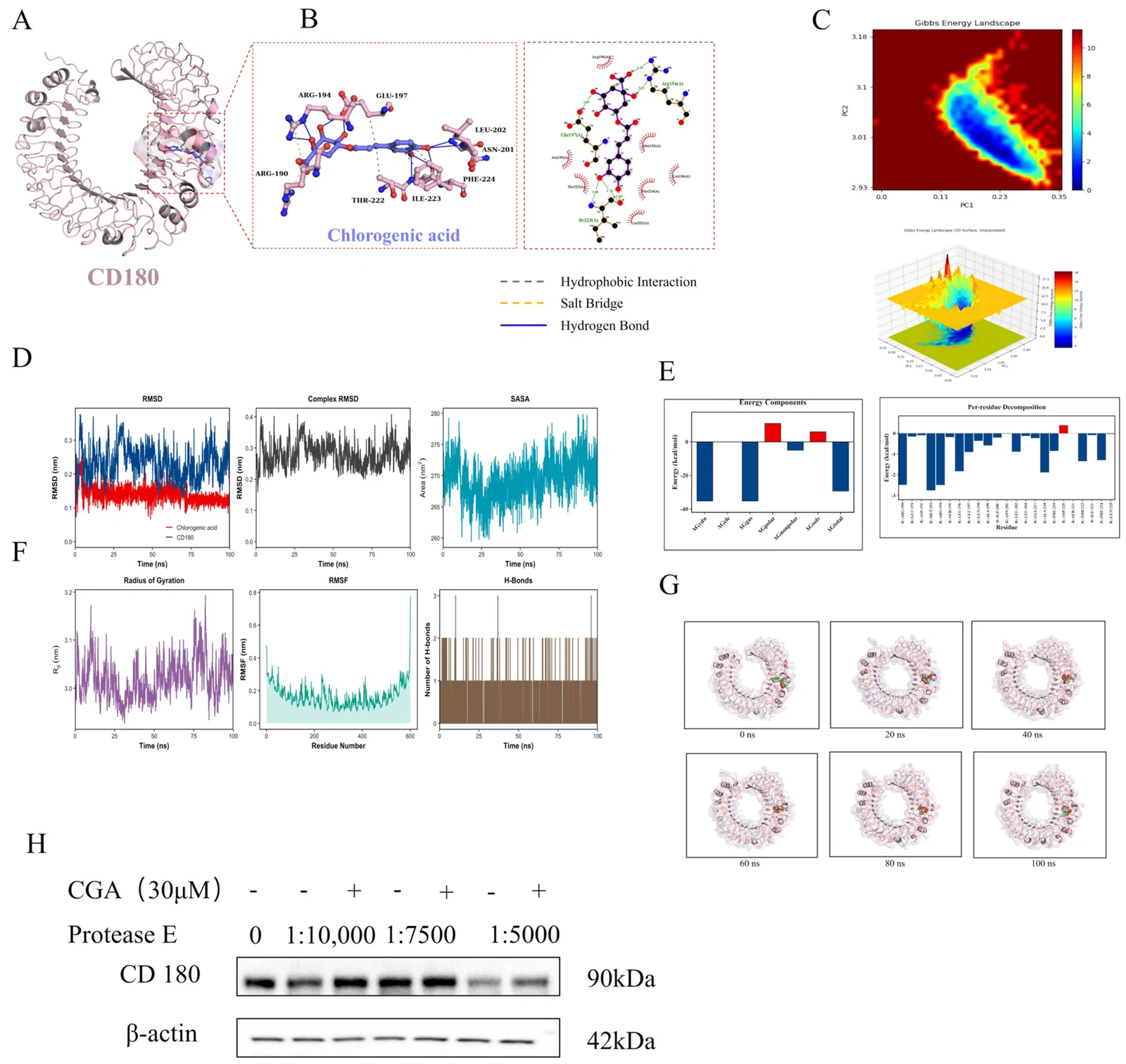
Molecular docking, molecular dynamics simulation, and exploratory DARTS analysis of the potential association between CGA and CD180. (**A**) Overall 3D predicted binding model of CGA with the CD180 protein. (**B**) 2D interaction map showing the predicted key residues involved in hydrogen-bonding, hydrophobic, and electrostatic interactions. (**C**) Gibbs free-energy landscape illustrating the conformational distribution of the CGA–CD180 complex during molecular dynamics simulation. (**D**) RMSD profiles of the CD180 protein (blue) and the CGA ligand (red) over a 100 ns simulation. (**E**) SASA and Rg of the complex. (**F**) RMSF of residue fluctuations and the number of hydrogen bonds formed during the simulation. (**G**) Representative structural snapshots of the complex at 0, 20, 40, 60, 80, and 100 ns. (**H**) Exploratory DARTS assay assessing CGA-associated changes in the proteolytic susceptibility of CD180 following incubation with CGA (30 μM) under the indicated protease conditions. β-actin was used as the loading control. This panel presents the CD180 blot and its corresponding matched β-actin loading control from the single experiment for which complete matched loading-control data were available. Therefore, this result is presented as a preliminary qualitative observation; reproducibility across independent experiments has not been established, and the DARTS result alone does not demonstrate direct or specific binding between CGA and CD180.

Molecular docking analysis was performed to evaluate the predicted interaction between CGA and CD180. The docking model suggested that CGA was positioned within a surface pocket of CD180 and formed hydrogen bonds and other non-covalent interactions with surrounding residues (Figure 11A,B).

Molecular dynamics simulations were further conducted to assess the stability of the predicted complex. During the 100 ns simulation period, the CD180–CGA complex showed relatively stable structural behavior. RMSD, RMSF, SASA, Rg, and hydrogen-bond analyses revealed limited fluctuations throughout the simulation (Figure 11C–F). Structural snapshots obtained at different time points indicated that CGA remained within the predicted binding region during the simulation (Figure 11G).

To further examine the potential association between CGA and CD180, DARTS analysis was performed. CD180 showed an apparent increase in resistance to protease digestion following CGA treatment compared with the untreated group, indicating a CGA-associated change in proteolytic stability (Figure 11H).

Collectively, the computational analyses and DARTS findings support a potential association between CGA and CD180.

### 2.12. CD180 Expression and MIN6 Insulin-Secretory Responses Following CGA Pretreatment of B Cells

The effects of CGA on CD180 expression and β-cell function were examined in the B cell–MIN6 co-culture system.

**Figure 12:**
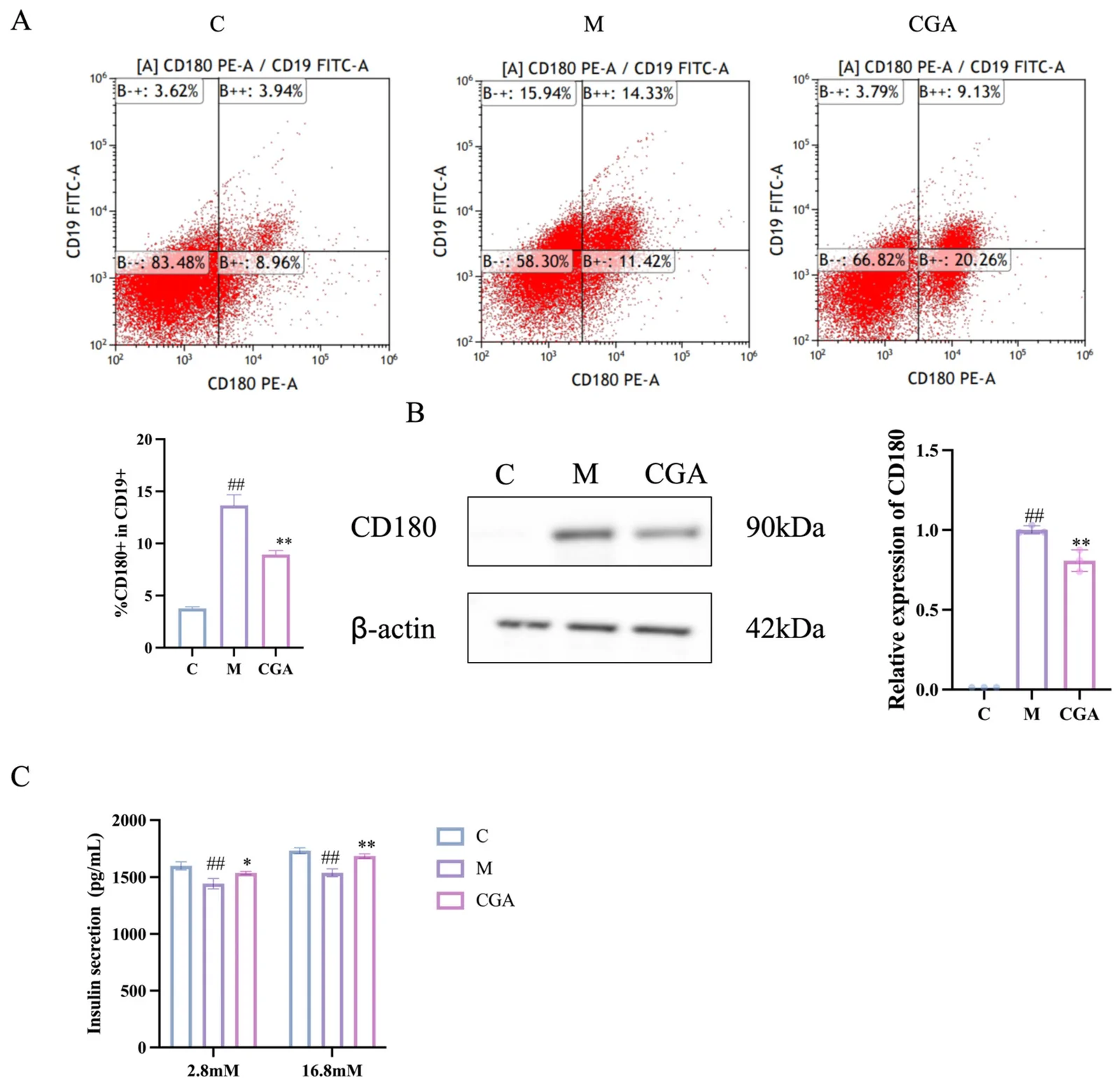
CD180 expression and MIN6 insulin-secretory responses following CGA pretreatment of B cells. (**A**) Representative flow-cytometry plots of the Control (C, left), Model (M, middle), and CGA (right) groups, with quantitative analysis of the percentage of CD180+ cells within the CD19+ B-cell population shown below (**B**) Western blot images (**left**) and densitometric quantification (**right**) of CD180 protein expression. (**C**) GSIS evaluated under 2.8 mM and 16.8 mM glucose conditions across the three groups. C, Control group; M, Model group; CGA, chlorogenic acid-treated group. Note: Data are presented as mean ± SD, *n* = 3. (^##^ *p* < 0.01 vs. Control group; * *p* < 0.05, ** *p* < 0.01 vs. Model group). Flow cytometry and Western blot data were analyzed using one-way ANOVA followed by Dunnett’s multiple-comparisons test. GSIS data were analyzed separately at 2.8 and 16.8 mM glucose using one-way ANOVA followed by Dunnett’s multiple-comparisons test. ## *p* < 0.01 vs. Control group; * *p* < 0.05 and ** *p* < 0.01 vs. Model group.

Flow cytometry showed increased CD180^+^ B-cell proportions after inflammatory stimulation, while CGA treatment reduced CD180-positive cells compared with the model group (Figure 12A and Figure S4). Western blot analysis confirmed increased CD180 protein expression after stimulation and its reduction following CGA treatment (Figure 12B).

MIN6 insulin secretion under the tested glucose conditions was higher following co-culture with CGA-pretreated B cells than with model-group B cells (Figure 12C).

These results indicate that CGA treatment is associated with reduced CD180 expression in inflammatory B cells and improved β-cell functional responses in vitro.

## 3. Discussion

We integrated transcriptomic datasets, single-cell profiling, and experimental validation to investigate B-cell-associated alterations in T1DM. CD180 was identified as a candidate molecule consistently associated with disease-related B-cell changes across multiple analytical approaches. Single-cell analysis showed that *Cd180* expression increased along the inferred inflammatory trajectory of B cells and was associated with transcriptional programs related to antigen processing and presentation. Structure-based screening further identified CGA as a potential CD180-associated compound. These findings show that CGA-associated changes in CD180 proteolytic stability and expression occurred alongside improved β-cell functional responses, supporting further investigation of the relationship between CD180-associated B-cell alterations and β-cell dysfunction in T1DM. These findings provide a basis for further investigating B-cell-related regulatory mechanisms in autoimmune diabetes.

Although T-cell-mediated mechanisms are well established in T1DM, accumulating evidence has expanded the role of B cells beyond antibody production. B cells can capture β-cell-derived antigens, present peptides through MHC class II molecules, and provide signals that influence autoreactive T-cell responses [15]. Studies in NOD mice have shown that B-cell deficiency reduces diabetes development, whereas restoration of antigen-presenting B cells promotes disease progression. Clinical observations are also consistent with a functional contribution of B cells, as B-cell depletion therapy transiently preserved C-peptide secretion in individuals with recent-onset T1DM [16]. However, the limited durability of this effect indicates that complete B-cell depletion may not be sufficient for sustained immune regulation. These findings highlight the need to identify molecular features that define disease-associated B-cell states and may provide opportunities for more selective immune intervention.

In our study, integrated analyses identified CD180 as a candidate marker associated with T1DM-related B-cell alterations. CD180 is a leucine-rich repeat-containing receptor mainly expressed on B cells and antigen-presenting cells and forms a functional complex with MD-1 [17]. Single-cell analysis showed that *Cd180* expression increased along the inferred transition of B cells toward an inflammatory state. In addition, computational perturbation analysis indicated that *Cd180*-associated transcriptional changes were enriched in antigen-presentation-related pathways. These findings suggest that CD180 may represent a molecular feature associated with altered B-cell immune characteristics in T1DM.

It should be noted that CD180 expression does not necessarily indicate a pathogenic B-cell phenotype. Although CD180 was consistently associated with inflammatory B-cell characteristics in our analyses, we cannot conclude that CD180 itself initiates autoimmune activation. An alternative interpretation is that CD180 may reflect a broader inflammatory B-cell state associated with altered activation and antigen-presenting properties. Further functional studies are needed to determine whether CD180 has a direct role in these changes or mainly serves as a marker of B-cell dysregulation.

Our single-cell and functional analyses together support a link between altered B-cell states and β-cell dysfunction in T1DM. The single-cell dataset showed that B-cell populations were heterogeneous and exhibited different immune characteristics, with potential interactions between immune and endocrine cells. These observations were consistent with the co-culture experiments, in which inflammatory B cells were associated with reduced MIN6 viability and impaired glucose-stimulated insulin secretion. The mechanisms connecting B-cell alterations with β-cell dysfunction are likely complex. Multiple factors, including inflammatory cytokines, antigen-presentation signals, and other immune mediators, may contribute to this process. It is also important to recognize that B cells include both inflammatory and regulatory subsets. For example, some B-cell populations can produce IL-10 and exert immunomodulatory effects. Therefore, increased CD180 expression may reflect a specific disease-associated B-cell state rather than universally indicating pathogenic B-cell activity.

An additional question raised by our study is whether CGA can modify the CD180-associated immune phenotype. CGA has been widely studied because of its antioxidant and metabolic activities [18,19], but its effects on specific immune-cell states are less clear. Here, CGA was selected from structure-based screening as a candidate compound related to CD180. The docking model and molecular dynamics simulation suggested a potential association between CGA and CD180, and the DARTS assay further showed changes in CD180 proteolytic stability after CGA treatment. Inflammatory B cells exposed to CGA showed reduced CD180 expression, accompanied by improved β-cell functional responses in the co-culture model. These observations do not indicate that CGA acts exclusively through CD180, as CGA can affect multiple cellular pathways. Rather, the changes observed in CD180-associated B-cell characteristics should be interpreted as part of the broader biological response to CGA rather than evidence of a CD180-specific mechanism.

The use of MSA-2 in NOD mice warrants specific consideration. Although spontaneous NOD diabetes provides an established model of autoimmune disease progression, its variable and relatively prolonged time course was not compatible with the predefined observation period of the present study. The selection of MSA-2 was informed by unpublished preliminary observations in our laboratory suggesting the earlier emergence of diabetes-related phenotypes following treatment, allowing exploratory assessment of metabolic and B-cell-associated changes within a defined experimental period. MSA-2 was not selected on the basis of evidence that it is superior to cyclophosphamide-induced acceleration, and no direct comparison among these approaches was performed in the present study. Rather, the choice reflected the specific experimental timeframe and our preliminary experience with this regimen. Accordingly, the present study does not establish MSA-2 as a generally applicable diabetes-acceleration strategy. Importantly, MSA-2 activates STING and can elicit broad innate immune responses, including type I interferon signaling. Consequently, the observed B-cell alterations and changes in CD180 may partly reflect treatment-associated immune perturbation and cannot be attributed specifically to B-cell/CD180 signaling. The in vivo findings should therefore be interpreted within this pharmacologically perturbed experimental setting and should not be assumed to reproduce the natural course of spontaneous autoimmune diabetes. Further studies using spontaneous NOD progression and additional experimental models will be required to determine the generalizability of these findings.

Several limitations of this study should be acknowledged. Although resampling-based internal assessment was performed, the selected predictors and upstream preprocessed expression matrix were fixed before resampling; therefore, independent external validation in additional T1DM cohorts remains necessary. CD180 showed consistent associations with T1DM-related B-cell alterations across bulk and single-cell transcriptomic analyses; whether CD180 directly contributes to these B-cell phenotypes remains unresolved. Functional studies using CD180 loss- and gain-of-function approaches will be required to determine its causal role. Second, although the computational analyses and DARTS findings support a potential association between CGA and CD180, these approaches do not establish direct or specific molecular binding. Further biophysical and functional validation will therefore be necessary to characterize this potential interaction. Third, CGA was evaluated only in the in vitro B cell–MIN6 co-culture system, and the animal experiments did not include a CGA-treated group. Accordingly, the present study does not establish the therapeutic efficacy of CGA in vivo, and the effects observed in vitro should not be directly extrapolated to an intact organism. Finally, the use of MSA-2-treated NOD mice represents an experimentally perturbed inflammatory setting rather than the natural progression of spontaneous autoimmune diabetes. Because MSA-2 activates STING and may induce broad innate immune and type I interferon responses, some of the observed B-cell and CD180 alterations may be influenced by STING-associated immune effects. Validation in spontaneously diabetic NOD mice and other experimental models will therefore be necessary to determine the generalizability of these findings. The DARTS result should be interpreted as a preliminary qualitative observation. Although three independent DARTS experiments were performed, matched β-actin loading-control data were available for only one experiment; therefore, Figure 11H presents the matched CD180 and β-actin blots from that experiment only. The absence of matched loading-control data for the other two experiments precludes assessment of reproducibility across independent biological experiments. Additional DARTS experiments with complete matched loading controls are required to validate this observation. Moreover, DARTS alone does not establish direct or specific molecular binding between CGA and CD180, which will require confirmation using orthogonal biophysical approaches.

Our data identify CD180-associated changes as a feature of T1DM-related B-cell alterations. The integration of transcriptomic, single-cell, and experimental analyses suggests that *Cd180* is linked to inflammatory B-cell states and antigen-presentation programs. In vitro, prior CGA exposure of inflammation-stimulated B cells was associated with reduced CD180 expression and improved MIN6 insulin-secretory responses during subsequent co-culture in CGA-free medium. Further work is needed to determine whether CD180 directly regulates these B-cell phenotypes and how CGA produces its immunomodulatory effects. Nevertheless, these findings provide new insight into the contribution of specific B-cell states to β-cell dysfunction in T1DM.

## 4. Materials and Methods

### 4.1. Bulk Transcriptomic Data Preprocessing and Integration

Transcriptomic datasets were obtained from the Gene Expression Omnibus (GEO) database. GSE156035 and GSE55098 were used to analyze peripheral blood mononuclear cell (PBMC) gene-expression profiles from individuals with T1DM and healthy controls. The single-cell RNA-sequencing dataset GSE141786 was analyzed separately to investigate B-cell heterogeneity and T1DM-associated transcriptional alterations.

The bulk-transcriptomic reanalysis was performed using R (v4.4.2) and Bioconductor (v3.20). GSE156035 and GSE55098 were assembled into a combined discovery cohort comprising 62 samples, including 20 controls and 20 T1DM cases from GSE156035 and 10 controls and 12 T1DM cases from GSE55098. Sample annotations and the preprocessing status of each expression matrix were checked against the corresponding GEO records before integration to avoid redundant normalization or log2 transformation. Where additional between-array normalization was required, quantile normalization was performed separately within each dataset using the normalizeBetweenArrays function in the limma package (v3.62.0).

Probe-level identifiers were mapped to gene symbols according to the corresponding platform annotation files. Unannotated probes and probes with ambiguous gene assignments were excluded. When multiple probes mapped to the same gene, their expression values were averaged to obtain a single gene-level expression value. Only genes shared between GSE156035 and GSE55098 were retained for subsequent dataset integration.

To reduce dataset-specific technical variation after integration, batch effects between GSE156035 and GSE55098 were adjusted using the ComBat function in the sva R package, with dataset identity specified as the batch variable. Principal component analysis (PCA) was performed before and after batch correction to assess dataset-associated variation and the effect of batch adjustment. The batch-corrected expression matrix was subsequently used for differential-expression analysis, weighted gene co-expression network analysis (WGCNA), and downstream candidate-gene analyses.

### 4.2. Weighted Gene Co-Expression Network Analysis

Weighted gene co-expression network analysis (WGCNA) was performed using the WGCNA R package (v1.73) to identify gene co-expression modules associated with T1DM [20]. Genes and samples were initially assessed using the goodSamplesGenes function. To reduce the influence of low-variability genes, genes in the upper quartile of median absolute deviation (MAD) were retained for network construction independently of disease-group labels. Hierarchical clustering was subsequently performed to assess sample relationships and identify potential outliers. The soft-thresholding power was evaluated using the pickSoftThreshold function based on scale-free topology fit and mean connectivity, and a soft-thresholding power of β = 8 was selected for network construction. An unsigned weighted gene co-expression network based on Pearson correlation was constructed using the blockwiseModules function with the following parameters: TOMType = “unsigned”, minModuleSize = 30, deepSplit = 2, mergeCutHeight = 0.25, pamRespectsDendro = FALSE, and maxBlockSize = 25000. The resulting adjacency matrix was transformed into a topological overlap matrix (TOM), and gene modules were identified by hierarchical clustering based on TOM dissimilarity followed by dynamic tree cutting. Module eigengenes were calculated to summarize the overall expression profile of each module and were correlated with T1DM status, coded as 0 for control samples and 1 for T1DM samples. *p* values for module–trait correlations were adjusted for multiple comparisons using the Benjamini–Hochberg method. Non-grey modules with adjusted *p* values < 0.05 were considered significantly associated with T1DM and were subsequently intersected with the differentially expressed genes to identify candidate genes.

### 4.3. Identification of Differentially Expressed Genes

Differential expression analysis between T1DM and control groups was performed using GEO2R. Genes with an adjusted *p* value <0.05 were selected as DEGs for downstream analyses.

### 4.4. Functional Enrichment Analysis

We used the clusterProfiler R package to perform GO and KEGG enrichment analyses. DEGs were submitted for functional annotation, including biological processes, cellular components, molecular functions, and pathway enrichment.

### 4.5. Machine Learning-Based Identification of Candidate Genes and Internal Model Assessment

We applied LASSO regression to narrow the initial gene set [21]. Random forest analysis was then used to examine the importance of the remaining genes [22]. Based on these results, we constructed an ANN model with the selected features [23]. ROC curves and AUC values were used to examine their ability to distinguish T1DM from control samples.

LASSO logistic regression was performed using the glmnet package (v4.1-8) with family = “binomial”, alpha = 1, and standardize = TRUE. The regularization parameter was selected using stratified 10-fold cross-validation with binomial deviance as the optimization criterion, and genes with nonzero coefficients at lambda.1se were retained. Random forest analysis was subsequently performed using the randomForest package (v4.7-1.1) with ntree = 1000, mtry = max(1, floor(sqrt(*p*))), and nodesize = 1, where *p* denotes the number of candidate predictors. Permutation-based variable importance was used to provide complementary rankings of the candidate genes. These feature-selection analyses were performed using the combined discovery cohort.

An exploratory artificial neural network (ANN) was implemented using MLPClassifier in scikit-learn (v1.5.2) under Python (v3.11.9). The network comprised one hidden layer containing five neurons and was configured with activation = “tanh”, solver = “lbfgs”, alpha = 0.01, and max_iter = 5000. These parameters were prespecified rather than optimized through hyperparameter tuning. The ANN was used as an exploratory classifier and was not included in the subsequent internal assessment.

To examine the stability of candidate-gene selection, LASSO feature selection was additionally repeated across bootstrap resamples of the discovery cohort. For each candidate gene, the proportion of bootstrap resamples in which it was retained by LASSO was calculated as its selection frequency. This analysis was used to assess the resampling stability of the candidate genes rather than to define an additional independent gene-selection step.

For supplementary internal assessment, *CD180*, *ZXDA*, *STEAP4*, and *KCNJ2* were retained as fixed predictors in a logistic regression model using the preprocessed expression matrix of 62 samples. The model was implemented in scikit-learn using the liblinear solver with an L2 penalty, C = 10^6^, and max_iter = 5000. Model-fitting optimism was estimated using 1000 bootstrap resamples. Within each bootstrap resample, predictor standardization parameters and regression coefficients were re-estimated, and model performance was evaluated in both the bootstrap sample and the original cohort. Optimism was calculated as the mean difference between these AUC estimates, and the mean optimism was subtracted from the apparent AUC to obtain an optimism-corrected estimate.

A complementary internal assessment was performed using stratified 10-fold cross-validation repeated 20 times. In each split, predictor standardization and model fitting were performed exclusively within the training folds, and the fitted model was subsequently applied to the held-out fold. AUC was calculated for each repetition. Held-out predicted probabilities for each sample were averaged across repetitions to generate a summary ROC curve and calculate the Brier score. A descriptive calibration plot was constructed using six quantile-based groups. The base random seed was set to 20260906.

Because the four predictors and the upstream preprocessed expression matrix were fixed before model resampling, these procedures represent a conditional internal assessment of the prespecified four-gene logistic model. They do not fully account for potential optimism introduced during the preceding feature-selection and batch-adjustment procedures and therefore should not be interpreted as independent external validation.

### 4.6. Protein–Protein Interaction Network Analysis

Protein–protein interaction (PPI) networks were generated using the STRING database based on the candidate genes. The networks were visualized in Cytoscape software (version 3.10.5), and densely connected modules were detected with the MCODE plugin.

### 4.7. Immune Infiltration Analysis

We applied the CIBERSORT algorithm to estimate immune cell fractions from transcriptomic expression matrices. The relationships between candidate genes and inferred immune cell proportions were assessed using correlation analysis [24].

### 4.8. Gene Set Enrichment Analysis

We performed GSEA using clusterProfiler R package (version 4.20.0) [25]. Genes were ranked according to their log2 fold-change values and analyzed against BIOCARTA gene sets obtained from the Molecular Signatures Database (MSigDB). Statistical significance was assessed using 1000 permutations. Gene sets with *p* < 0.05 and false discovery rate (FDR) <0.25 were considered significantly enriched.

### 4.9. Isolation of Primary B Cells and Establishment of the Co-Culture System

Splenic B cells were isolated from 6-week-old C57BL/6J mice using magnetic bead-based negative selection. After mechanical dissociation and filtration, CD45R^+^ cells were purified according to the manufacturer’s protocol. Purity was assessed by flow cytometry.

Primary B cells were simultaneously stimulated with LPS (20 μg/mL), IFN-γ (20 ng/mL), and IL-1β (10 ng/mL) for 48 h. In the CGA group, CGA (30 μM) was added at the start of stimulation and remained present throughout this period. After treatment, B cells in the model and CGA groups were collected by centrifugation; the supernatants were discarded, and the cells were washed three times with PBS. B cells from each group were counted using an automated cell counter before co-culture. MIN6 cells were then co-cultured in direct contact with B cells at a 1:20 ratio for 48 h in fresh CGA-free 1640 medium. No CGA was added during co-culture. The control group consisted of unstimulated B cells co-cultured with MIN6 cells. The model group consisted of B cells that were stimulated with inflammatory agents, washed, and then co-cultured with MIN6 cells, whereas the CGA group consisted of B cells exposed to CGA during inflammatory stimulation, washed, and subsequently co-cultured with MIN6 cells.

### 4.10. Transcriptomic Sequencing and Analysis

After co-culture with MIN6 cells, B cells were collected for RNA extraction. RNA sequencing was performed on the control group (unstimulated B cells co-cultured with MIN6 cells) and the model group (inflammatory-stimulated B cells co-cultured with MIN6 cells). Each group contained three independent biological samples. RNA-seq libraries were prepared and sequenced using the Illumina NovaSeq 6000 platform after quality assessment.

Sequencing reads underwent quality control before expression quantification with featureCounts (v2.0.3). Differential expression analysis was performed using DESeq2 (v1.38.3). Genes meeting the criteria of |log2(fold change)| ≥1 and adjusted *p* value < 0.05 were selected as DEGs.

### 4.11. Construction of Regulatory Networks

TF–gene associations were retrieved from the hTFtarget database to explore potential transcriptional regulation of candidate genes [26]. We collected miRNA–mRNA interaction data from miRanda, TargetScan, and miRDB. Only overlapping miRNA targets identified by multiple databases were retained for further analysis.

The miRNA–lncRNA and TF–lncRNA regulatory relationships were obtained from miRNet, starBase, LncBase v3.0, and LncACTdb 3.0. The integrated regulatory networks were constructed and visualized using Cytoscape.

### 4.12. Single-Cell RNA Sequencing Analysis and Virtual Gene Perturbation

Single-cell RNA sequencing data from GSE141786 were analyzed using the Seurat package (v5.0.1) [27]. Processed gene–barcode expression matrices corresponding to the 4-, 8-, and 15-week time points were imported into R. The input matrices used in the present reanalysis contained 52,164 cells before quality-control filtering.

For quality control, genes detected in fewer than three cells were excluded during construction of the Seurat objects (min.cells = 3). At the cell level, cells with fewer than 200 or more than 2500 detected genes or with mitochondrial transcript proportions ≥20% were excluded. Mitochondrial transcript percentages were calculated based on genes beginning with “MT-”. Following quality-control filtering, 46,734 cells were retained for subsequent analysis. Gene-expression matrices were normalized using the NormalizeData function with the LogNormalize method and a scale factor of 10,000. The top 2000 highly variable genes were identified using the FindVariableFeatures function with the vst method, followed by data scaling using ScaleData and principal component analysis (PCA).

Harmony was applied to reduce technical variation among samples, with sample identity specified as the batch variable. The first 30 Harmony-corrected dimensions were used for nearest-neighbor graph construction, clustering, and UMAP visualization. Cell clusters were annotated using canonical cell-type-specific marker genes. Following cell-type annotation, 17,354 B cells were selected for downstream analysis, including 8324 cells at 4 weeks, 7381 cells at 8 weeks, and 1649 cells at 15 weeks. The β-cell population included 836 cells, comprising 483 Beta0, 326 Beta1, and 27 Beta2 cells. These counts represent the B-cell and β-cell populations selected for their respective downstream analyses and are distinct from the total of 46,734 cells retained after quality-control filtering. B cells were analyzed to characterize Cd180-associated transcriptional alterations and inflammatory states, whereas β cells were examined to investigate predicted communication patterns between immune and endocrine cell populations.

Inflammatory-response-associated transcriptional patterns were evaluated at the single-cell level using the HALLMARK_INFLAMMATORY_RESPONSE gene set obtained from the Molecular Signatures Database (MSigDB). Genes that were not detected in the single-cell expression matrix or could not be reliably mapped to mouse orthologues were excluded before score calculation. Inflammatory-response scores were calculated from the log-normalized RNA expression matrix using the Seurat AddModuleScore function. Genes were divided into 24 bins according to their average expression levels (nbin = 24), and 100 control genes (ctrl = 100) with comparable average expression levels were randomly selected for each analyzed feature. For each cell, the inflammatory-response score represented the average expression of genes in the inflammatory-response signature relative to that of the matched control genes. Thus, higher scores indicated relatively greater expression of the inflammatory-response gene signature rather than absolute activation of an inflammatory pathway. Scores were compared among the relevant experimental groups and annotated cell populations and visualized using UMAP feature plots, violin plots, and box plots. For cell-level comparisons, differences between two groups were assessed using the Wilcoxon rank-sum test, whereas comparisons involving more than two groups were assessed using the Kruskal–Wallis test followed, where appropriate, by pairwise Wilcoxon rank-sum tests. All statistical tests were two-sided, and *p* < 0.05 was considered statistically significant.

Pseudotime trajectory analysis was performed for selected cell populations using Monocle 2. Genes expressed in at least 10 cells were retained for trajectory analysis. Genes showing differential expression among the relevant cell subsets were identified using differentialGeneTest, and the top 1000 genes ranked according to statistical significance were selected as ordering genes. Dimensionality reduction was performed using the DDRTree algorithm, followed by cell ordering along the inferred trajectory using the orderCells function. The root state was assigned to the transcriptionally less activated population based on canonical marker expression. Changes in *Cd180* expression and associated transcriptional states were subsequently examined along the inferred pseudotime trajectory.

Ligand–receptor-mediated intercellular communication was analyzed using CellChat and the curated CellChatDB.mouse database. CellChat objects were constructed using normalized expression matrices and corresponding cell-type annotations. Overexpressed signaling genes and ligand–receptor interactions were identified using identifyOverExpressedGenes and identifyOverExpressedInteractions, respectively. Communication probabilities were estimated using computeCommunProb, and cell populations containing fewer than 10 cells were excluded from subsequent communication analyses. Pathway-level communication probabilities were calculated using computeCommunProbPathway, followed by network aggregation using aggregateNet. Ligand–receptor interactions with permutation-derived *p* values < 0.05 were considered statistically significant.

In silico perturbation analysis of *Cd180* was performed using the scTenifoldKnk package (v1.0.2) [28] on the raw count matrix of the selected cell population. Ten subsampled single-cell gene regulatory networks were constructed using principal component regression, with 500 cells sampled per network and three principal components (nc_nNet = 10, nc_nCells = 500, and nc_nComp = 3). Network construction was performed with nc_scaleScores = TRUE, nc_symmetric = FALSE, nc_lambda = 0, and an edge-filtering parameter of nc_q = 0.9. The resulting networks were denoised using rank-three tensor decomposition with a maximum of 1000 iterations and a convergence tolerance of 10^−5^. In silico perturbation of *Cd180* was simulated by setting its outgoing regulatory edges to zero. Two-dimensional manifold alignment was subsequently used to compare the original and perturbed gene regulatory networks and identify genes associated with computational *Cd180* perturbation. These genes were subsequently subjected to Gene Ontology (GO) and Kyoto Encyclopedia of Genes and Genomes (KEGG) enrichment analyses.

### 4.13. Animal Model and Experimental Design

Female NOD mice (8 weeks old) were obtained from Suzhou Hekai Biotechnology Co., Ltd. (Suzhou, China). The animals were maintained under specific pathogen-free conditions with free access to food and water.

MSA-2, a STING agonist, was used to examine metabolic and B-cell-associated changes following pharmacological immune perturbation in NOD mice. This treatment was not intended to selectively manipulate B cells or CD180, but rather to provide an experimentally perturbed immune setting within the observation period. Mice in the MSA-2 group received subcutaneous injections of MSA-2 at 20 mg/kg on days 0 and 14. Vehicle-treated mice received an equivalent volume of vehicle by subcutaneous injection at the corresponding time points.

Body weight and fasting blood glucose levels were recorded throughout the study. At the end of the experiment, blood samples were collected for insulin and C-peptide analysis, and pancreatic tissues were harvested for histological evaluation.

All animal experiments were reviewed and approved by the Institutional Animal Care and Use Committee (Approval No. SZHK2024017).

### 4.14. Western Blot Analysis

We extracted proteins from cells or pancreatic tissues using RIPA buffer with protease inhibitors. Protein concentrations were determined by the BCA assay.

The same amount of protein from each sample was resolved by SDS-PAGE and transferred to PVDF membranes. Membranes were blocked and incubated with primary antibodies overnight at 4 °C.

Following secondary antibody incubation, target proteins were visualized using an enhanced chemiluminescence system.

### 4.15. Cell Viability Assay

MIN6 cells were seeded into 96-well plates and co-cultured with stimulated B cells. After treatment, non-adherent cells were removed by washing with PBS. MIN6 cell viability was then measured using the CCK-8 assay. Absorbance at 450 nm was recorded with a microplate reader.

### 4.16. Glucose-Stimulated Insulin Secretion Assay

MIN6 cells were washed after treatment and equilibrated in KRBH buffer containing 0.1% BSA for 1 h. GSIS was assessed by incubating cells with a 2.8 mM or 16.8 mM glucose buffer for 1 h. Supernatants were collected for insulin measurement by ELISA.

### 4.17. Flow Cytometry

We analyzed CD180 expression by flow cytometry after collecting and staining the cells. Lymphocytes were first selected according to FSC/SSC characteristics, and doublets were removed before identifying CD19^+^ B cells. CD180 expression was determined by calculating the proportion of CD180^+^ cells within the CD19^+^ B-cell population.

### 4.18. Virtual Screening and Molecular Dynamics Simulation

The CD180 structure (PDB ID: 3B2D) was downloaded from the RCSB Protein Data Bank. Virtual screening was performed using the Glide module in Schrödinger Suite (Release 2026-3). Compounds were evaluated using HTVS, SP, and XP docking protocols [29].

Molecular dynamics simulations were conducted with GROMACS using the CHARMM36 force field. The protein–ligand complexes were prepared in TIP3P solvent, neutralized with ions, and energy minimized before NVT/NPT equilibration and a 100 ns production simulation.

Structural stability and ligand interactions were analyzed based on RMSD, RMSF, Rg, SASA, hydrogen bonding, and MM/PBSA binding energy estimation.

### 4.19. DARTS Assay

B-cell lysates were incubated with CGA (30 μM) or vehicle control. After ligand treatment, samples were subjected to Pronase digestion. The reactions were stopped by adding loading buffer and heating at 95 °C. Changes in CD180 proteolytic stability were analyzed by Western blotting.

### 4.20. Statistical Analysis

Statistical analyses were performed using GraphPad Prism 8.0. Quantitative data are presented as the mean ± standard deviation. Normality of the data and homogeneity of variances were assessed using the Shapiro–Wilk test and Levene’s test, respectively. For comparisons between two independent groups, an unpaired two-tailed Student’s *t*-test was used when the assumptions of normality and homogeneity of variance were satisfied. For comparisons involving more than two independent groups, one-way analysis of variance followed by Dunnett’s multiple-comparisons test was performed when comparisons with a designated control or model group were required. Longitudinal measurements of body weight and fasting blood glucose in the same animals were analyzed using two-way repeated-measures ANOVA, with treatment group and time as factors, followed by Šídák’s multiple-comparisons test where appropriate. For GSIS experiments involving multiple treatment groups, statistical analyses were performed separately at each glucose concentration using one-way ANOVA followed by Dunnett’s multiple-comparisons test. For GSIS experiments involving only two groups, comparisons were performed separately at each glucose concentration using an unpaired two-tailed Student’s *t*-test. Unless otherwise specified, cell-based experiments were performed using three independent biological replicates (*n* = 3), whereas the animal experiments shown in Figure 8 included six mice per group (*n* = 6). Technical replicates were averaged within each independent biological replicate and were not treated as independent observations for statistical analysis. The exact sample size and statistical test used for each experiment are specified in the corresponding figure legends. A two-sided *p* value < 0.05 was considered statistically significant.

## 5. Conclusions

This study identified CD180-associated B-cell alterations as a disease-related feature of T1DM through integrated transcriptomic and experimental analyses. CD180 was associated with inflammatory B-cell states and antigen-presentation-related programs, whereas CGA treatment in vitro was accompanied by reduced CD180 expression in activated B cells and improved β-cell functional responses. These findings expand the understanding of B-cell-associated immune alterations in T1DM and provide a basis for further investigation of the relationship between CGA, CD180-associated B-cell states, and β-cell dysfunction.

## Data Availability

The transcriptomic datasets utilized in this study were obtained from the Gene Expression Omnibus (GEO) public database (https://www.ncbi.nlm.nih.gov/geo/, accessed on 13 January 2026). The GSE156035 dataset comprises the global gene expression profiles of peripheral blood mononuclear cells (PBMCs) derived from 20 T1DM patients positive for at least one islet autoantibody and 20 healthy controls negative for islet autoantibodies. The GSE55098 dataset, also originating from PBMCs of T1DM patients and normal controls, was employed to investigate the immunophenotypes, molecular regulatory mechanisms, and key gene expression signatures during T1DM progression. The single-cell dataset GSE141786 was downloaded from the GEO database.

## Supplementary Materials

The following supporting information can be downloaded at: https://www.mdpi.com/article/10.3390/ijms27198933/s1.

## Abbreviations

Type 1 diabetes (T1DM); Major Histocompatibility Complex (MHC); Non-obese diabetic (NOD); Regulatory B cell (Bregs); Chlorogenic acid (CGA); Gene Expression Omnibus (GEO); Peripheral blood mononuclear cells (PBMC); Soft-thresholding power (β); Topological Overlap Matrix (TOM); Differentially expressed genes (DEGs); Gene Ontology (GO); Biological process (BP); Cellular component (CC); Molecular function (MF); Kyoto Encyclopedia of Genes and Genomes (KEGG); Random Forest (RF); Artificial Neural Network (ANN); Protein–Protein Interaction (PPI).
