# Supplementary material for: CD180-Associated B-Cell Alterations in Type 1 Diabetes and the In Vitro Effects of Chlorogenic Acid: An Integrated Transcriptomic and Experimental Study
Source: Int J Mol Sci. 2026 Oct 8;27(19):8933. doi: 10.3390/ijms27198933 (PMC13666279; doi:10.3390/ijms27198933)
Supplement: Supplementary file 1 [file ijms-27-08933-s001.zip › ijms-4530458-supplementary.pdf]

International Journal of
Molecular Sciences

MDPI

Supplementary Materials

The stability of candidate-gene selection and the discriminatory performance of the four-gene signature were further evaluated using resampling-based internal assessment (Figure S1). The prespecified four-gene logistic regression model showed an apparent AUC of 0.883, whereas the summary ROC curve based on repeated stratified 10-fold cross-validation yielded an AUC of 0.827 (Figure S1A). Bootstrap resampling estimated a mean AUC optimism of 0.032 (Figure S1B), indicating a modest degree of model-fitting optimism. Cross-validated predictions yielded a Brier score of 0.174. The calibration plot showed an overall concordant trend between predicted and observed probabilities, although some deviation was evident at intermediate predicted probabilities (Figure S1C). Bootstrap LASSO analysis showed that CD180 and ZXDA were the most consistently selected candidate genes, whereas STEAP4 and KCNJ2 exhibited comparatively lower selection frequencies (Figure S1D). These findings provide conditional internal support for the stability and discriminatory performance of the four-gene model but do not constitute independent external validation. Figure S2: Additional flow-cytometry plots and event-count summaries for CD45, CD3, and CD19 analysis; Figure S3: Additional flow-cytometry plots for assessment of B-cell purity and CD19/CD180 staining, corresponding to Figure 9A and Figure 9F, respectively; Figure S4: Additional FSC-A/SSC-A, CD19/SSC-A, and CD180/SSC-A flow-cytometry plots corresponding to Figure 12A.

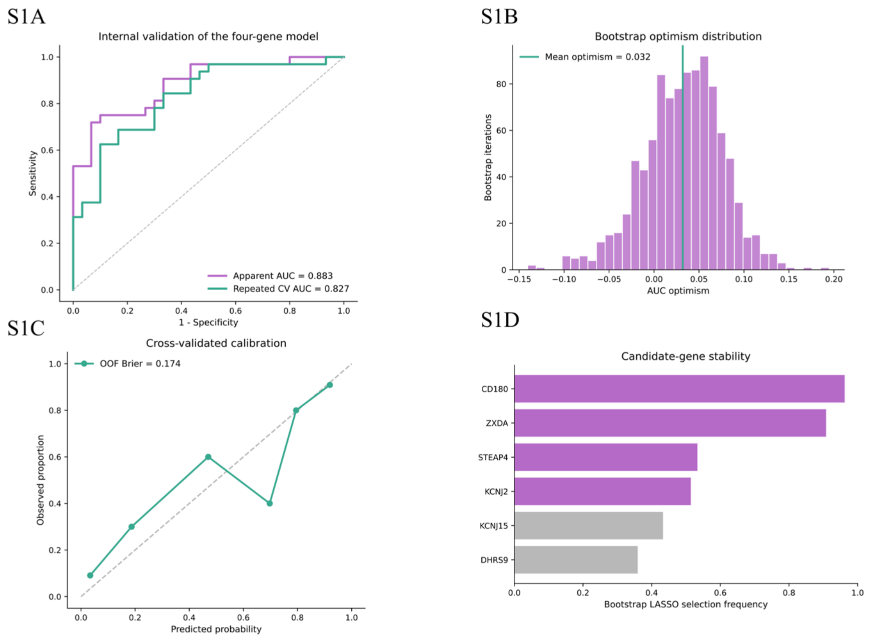

Figure S1. Resampling-based stability analysis and internal assessment of the four-gene model. (**A**) ROC curves showing the apparent discriminatory performance of the prespecified four-gene logistic regression model and the summary performance based on repeated stratified 10-fold cross-validated held-out predictions. (**B**) Distribution of AUC optimism estimated by bootstrap resampling; the vertical line indicates the mean optimism. (**C**) Descriptive calibration plot based on cross-validated predicted probabilities, with the corresponding out-of-fold Brier score shown. (**D**) Bootstrap LASSO selection frequencies of candidate genes, representing the proportion of bootstrap resamples in which each gene was retained. These analyses represent conditional internal assessment and do not constitute independent external validation.

S2

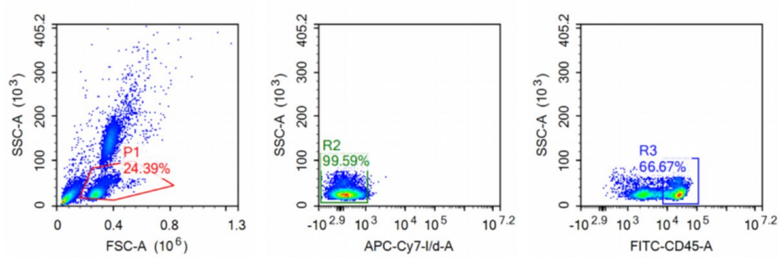

Figure S2. Representative flow-cytometry gating strategy used for immune-cell analysis. Representative plots illustrating the sequential gating procedure used to identify the cell populations analyzed in the study. Gates were applied sequentially as indicated in the panels. The percentages shown indicate the proportion of events within the corresponding parent population.

S3A

S3B

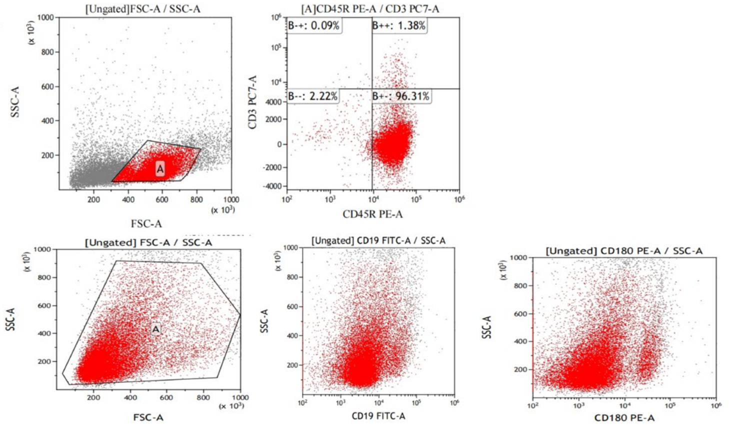

Figure S3. Flow-cytometric characterization of isolated primary B cells and CD180 expression under inflammatory stimulation. (**A**) Representative flow-cytometry analysis of primary B cells following isolation. CD45 and CD3 staining was used to characterize the isolated cell population and assess the enrichment of B cells prior to subsequent experiments. (**B**) Representative flow-cytometry plots showing CD19 and CD180 expression in the Control (C) and Model (M) groups. The Model group consisted of B cells subjected to inflammatory stimulation. Note: C, Control group; M, Model group.

S4

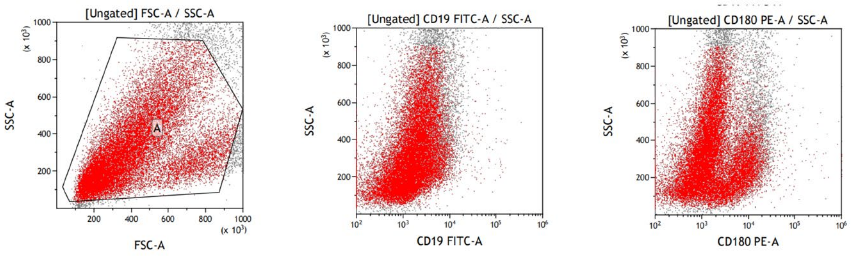

Figure S4. Representative flow-cytometry analysis of CD180 expression in B cells following CGA treatment. Representative flow-cytometry plots showing CD19 and CD180 expression in the Control (C), Model (M), and CGA-treated groups. B cells in the Model group were subjected to inflammatory stimulation, whereas B cells in the CGA group were exposed to CGA (30 μM) during inflammatory stimulation. Note: C, Control group; M, Model group; CGA, chlorogenic acid-treated group.

Disclaimer/Publisher's Note: The statements, opinions and data contained in all publications are solely those of the individual author(s) and contributor(s) and not of MDPI and/or the editor(s). MDPI and/or the editor(s) disclaim responsibility for any injury to people or property resulting from any ideas, methods, instructions or products referred to in the content.

https://doi.org/10.3390/ijms27198933
